# Disease mutations in striated muscle myosins

**DOI:** 10.1007/s12551-020-00721-5

**Published:** 2020-07-10

**Authors:** Francine Parker, Michelle Peckham

**Affiliations:** School of Molecular and Cellular Biology, Faculty of Biological Sciences, University of Leeds, Leeds, LS2 9JT UK

**Keywords:** β-Cardiac myosin, α-Cardiac myosin, Embryonic myosin, Cardiac disease, Skeletal muscle disease, Missense mutation

## Abstract

Over 1000 disease-causing missense mutations have been found in human β-cardiac, α-cardiac, embryonic and adult fast myosin 2a myosin heavy chains. Most of these are found in human β-cardiac myosin heavy chain. Mutations in β-cardiac myosin cause hypertrophic cardiomyopathy predominantly, whereas those in α-cardiac are associated with many types of heart disease, of which the most common is dilated cardiomyopathy. Mutations in embryonic and fast myosin 2a affect skeletal muscle function. This review provides a short overview of the mutations in the different myosin isoforms and their disease-causing effects.

**Electronic supplementary material:**

The online version of this article (10.1007/s12551-020-00721-5) contains supplementary material, which is available to authorized users.

## Introduction

Myosins are molecular motors, which bind actin and nucleotide, and use the chemical energy from ATP hydrolysis to drive movement. The human genome encodes 38 myosin genes, organised into 12 classes. The largest class of myosin is class 2 (Peckham 2016). Myosins in this class are comprised of two heavy chains, and four light chains, of which two are essential, and two are regulatory. The two heavy chains dimerise to form a long coiled-coil tail, which enables these proteins to assemble into filaments.

**Fig. 1:**
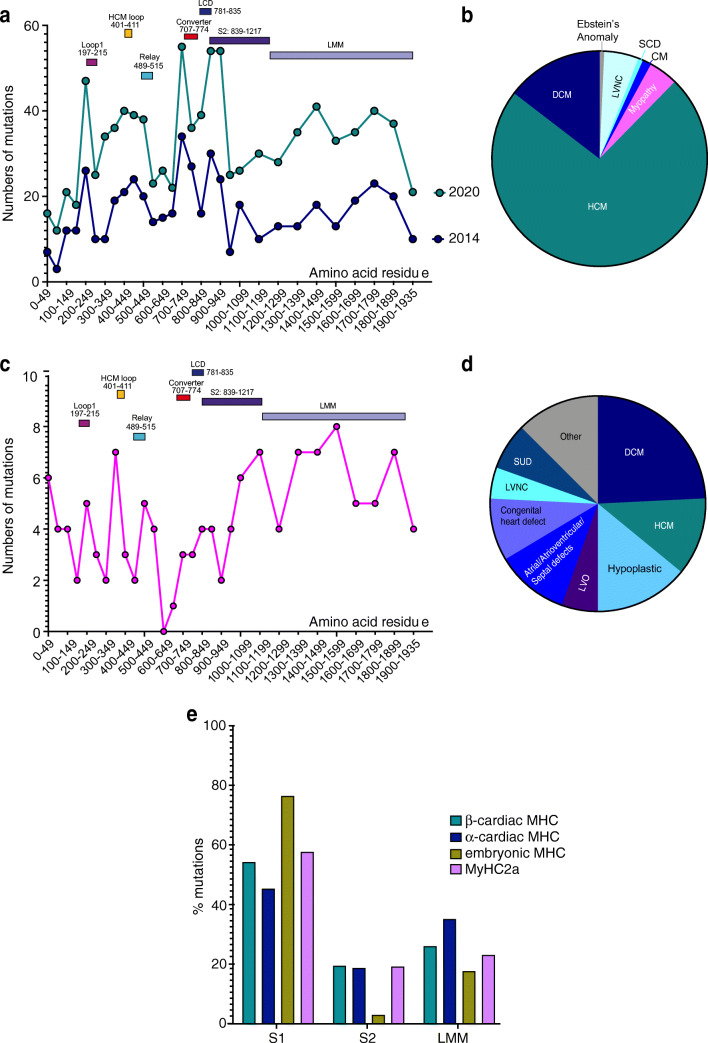
Analysis of myosin mutations in myosin heavy chains. **a** Graph showing the pattern of missense mutations that occur along the length of the β-cardiac myosin heavy chain amino acid sequence. Data shown includes mutations in this and an earlier study in 2014 with fewer mutations (Colegrave and Peckham 2014). The total numbers of mutations for each stretch of 50 amino acids (for subfragment-2 (S1) and subfragment-2 (S2)) and for 100 amino acid stretches (for light meromyosin (LMM)) are shown. Key functional regions of the motor are identified. LCD, light chain–binding domain. **b** Chart to indicate the main diseases resulting from mutations in β-cardiac myosin heavy chain (see also Supplementary Table 1). HCM, hypertrophic cardiomyopathy; DCM, dilated cardiomyopathy; CM, cardiomyopathy; SCD, sudden cardiac death; LVNC, left ventricular non-compaction. **c** Graph showing the pattern of missense mutations that occur along the length of the α-cardiac myosin heavy chain amino acid sequence, as in **a**. **d** Chart to indicate the main diseases resulting from mutations in α-cardiac myosin heavy chain (see also Supplementary Table 2). Abbreviations as in **b**. Additional abbreviations: SUD, sudden unexplained death; LVO, left ventricular obstruction. **e** Percentage of mutations in the three main regions of myosin, S1 (motor and lever), S2 and LMM for the 4 different myosin heavy chains

Striated muscle myosin isoforms are all class 2 myosins. One or more disease mutations have been described for these isoforms, with missense mutations most common. Four isoforms (β-cardiac, α-cardiac, embryonic and adult fast myosin 2a) are most commonly mutated. The most commonly mutated of these is β-cardiac myosin heavy chain (MYH7: Uniprot), which is expressed in both cardiac and in slow skeletal muscle and in developing muscle fibres (Schiaffino and Reggiani 2011). Over 1000 mutations have been reported, of which the majority (92%) are missense. In the adult heart, it is mainly found in the ventricles and mutations in the gene encoding this protein mainly cause hypertrophic cardiomyopathy (HCM) (Supplemental Table 1, Fig. 1). α-Cardiac myosin heavy chain (MYH6: uniprot) is the next most commonly mutated. It is almost exclusively expressed in the heart, where it is mainly found in the atria (Bouvagnet et al. 1989). Out of a total of 145 mutations, 128 are missense. These mutations are associated with HCM and dilated cardiomyopathy (DCM) and at least 6 other disease phenotypes in the heart (Supplementary Table 2, Fig. 1).

Two skeletal myosin heavy chains have also been reported to have more than 10 mutations. Embryonic myosin heavy chain (MYH3: Uniprot) is the first myosin isoform to be expressed in developing muscle fibres, is re-expressed in the early stages of muscle regeneration in adults and is expressed in some specialised adult muscles (Schiaffino et al. 2015). Twenty-six missense mutations in this gene have been reported, of which most are associated with distal arthrogryposis (Supplementary Table 3). Twenty-six missense mutations have been reported for myosin heavy chain 2A (MyHC2a: MYH2: Uniprot), which is found in fast skeletal muscle fibres (Type 2A) (Schiaffino et al. 2015). Mutations in its gene are associated with myopathies (Supplementary Table 4).

In this review, we have revisited our previous analysis of the positions of the known disease-causing mutations in the β-cardiac myosin heavy chain sequence (Colegrave and Peckham 2014) and extended it to α-cardiac, embryonic and adult fast myosin 2a. The clustering of the disease mutations into important functional regions in β-cardiac myosin heavy chain is similar to that of our earlier study. Mutations in α-cardiac, embryonic and adult fast myosin 2a, although much smaller in number, are also largely distributed throughout the sequence, and found in key functional regions.

## Myosin structure—a brief overview

The structure of myosin motor domains has been solved many times (e.g. see Houdusse and Sweeney (2016)) and is only reviewed briefly here. The motor and light chain–binding domains make up subfragment-1 (S1) of myosin (comprised of 25K, 50K and 20K subdomains (Rayment et al. 1993)). The N-terminal 25K domain comprises the N-terminal ‘SH3’ like fold, 6 of the 7 strands in the 7-stranded β-sheet core, and the ‘GESGAGKT’ motif for the well-conserved phosphate-binding loop at the base of the nucleotide-binding pocket. The junction between the 25K and central 50K domain lies within loop 1, just after the phosphate-binding loop. This domain is separated into two functional domains known as the upper and lower 50K domains. Two motifs, switch 1 and switch 2 (Geeves and Holmes 1999), respond to the binding of ATP in the nucleotide pocket by moving close together when ATP is bound, and further apart after phosphate is released, conveying information about the occupancy of the nucleotide pocket to the rest of the molecule.

On phosphate release, the cleft between the upper and lower 50K domains closes, the converter and light chain–binding domain undergo a large transition, such that the lever (mostly comprised of the LCD) swings by a large angle (Geeves and Holmes 1999). When bound to actin, it is this movement of the lever that drives movement of the actin filaments, and hence contraction. Loop 1, which lies above the nucleotide-binding pocket, may modulate the ATPase kinetics of the motor, and in particular the rate of ADP release (Uyeda et al. 1994). The actin-binding regions within myosin are mainly comprised of loops 2, 3 and 4, the HCM loop, the activation loop and the helix-loop-helix (von der Ecken et al. 2016).

The motor domain and lever are followed by an α-helical region that homodimerises to form a coiled coil, beginning at the invariant proline (residue 838, Fig. 1). The coiled coil has a characteristic 7 heptad amino acid residue repeat (***a****bc****d****efg*) in which the ‘a’ and ‘d’ residues tend to be buried in the hydrophobic seam. The first part of the coiled coil forms a region known as subfragment-2 (S2, here defined as residues 838-1217). The remainder of the coiled coil is known as light meromyosin (LMM) and is the filament forming region of the myosin heavy chain. Twenty-eight residue repeats of alternating charge enable the LMM to pack together into filaments with a defined stagger between each molecule (McLachlan and Karn 1982). Four ‘skip’ residues (Offer 1990) interrupt this regular repeat within the coiled coil and the structures of the coiled coil in these 4 regions were recently solved (Taylor et al. 2015).

It has recently become clear that myosin heads interact with each other to form the ‘interacting heads motif’ (IHM) in striated muscle isoforms, similar to that originally described to non-muscle and smooth muscle myosin (Wendt et al. 2001). In the IHM, in the filament, the two myosin heads are thought to interact with S2 (Alamo et al. 2017; Woodhead and Craig 2020), as well as with myosin-binding protein C (MyBPC (Spudich 2015). The formation of IHM is correlated with a low ATPase activity (McNamara et al. 2015), thus helping to conserve ATP usage by muscles when not in use. Activation of the muscle not only involves the canonical movement of tropomyosin on the thin filament to expose binding sites for myosin heads but also additionally requires myosin heads to be released from their shutdown state (Irving 2017). It is likely that phosphorylation of the regulatory light chains (RLCs) plays a role in this in both cardiac and skeletal muscles (Yu et al. 2016), through affecting the stability of the shutdown state.

## Mutations in β-cardiac myosin heavy chain and disease

Almost 1000 mutations in the gene that encodes β-cardiac myosin heavy chain have now been described (Supplementary Table 1). These mutations were retrieved from the professional version of the Human Gene Mutation Database (HGMD: (Stenson et al. 2017), an up-to-date version of the HGMD run by the Institute of Medical Genetics in Cardiff. Each mutation described in this database is curated from peer-reviewed publications, and classified as disease causing, or probable/possible disease causing based on this published evidence (see associated references in Supplementary Table 1). Other mutation databases are also available and are useful, such as ClinVar (Landrum et al. 2016) and the Leiden open variation database (LOVD: http://www.lovd.nl (Harrison et al. 2016). However, a significant number of mutations (~ 40%) in these databases are directly submitted and lack supporting evidence from a peer-reviewed publication, and should be viewed with more caution.

Although the number of mutations in the HGMD database has almost doubled compared with that reported 5 years ago (Colegrave and Peckham 2014), the pattern of mutations is similar (Fig. 1a). The majority (~ 58%) of the HCM mutations in β-cardiac myosin are found in the motor domain and lever (S1). Approximately 15% of mutations are found in S2 and the remaining mutations (27%) are found in LMM.

The majority (73%) of the missense mutations in β-cardiac myosin heavy chain cause HCM (Fig. 1b), and these mutations are found throughout the sequence (Fig. 1a). The first mutation to be linked to this disease was the R403Q mutation, which lies within a loop known as the HCM loop in the motor domain (Geisterfer-Lowrance et al. 1990). With so many mutations reported, there is little experimental work on most of them, and mutations identified may, in some cases, correlate with disease rather than being causal. HCM may affect up to 1 in 200 of the population (Semsarian et al. 2015), is the most common cause of sudden death in the under 30 age group, and mutations in β-cardiac myosin heavy chain are responsible for ~ 40% of cases of HCM (Maron 2002).

**Fig. 2:**
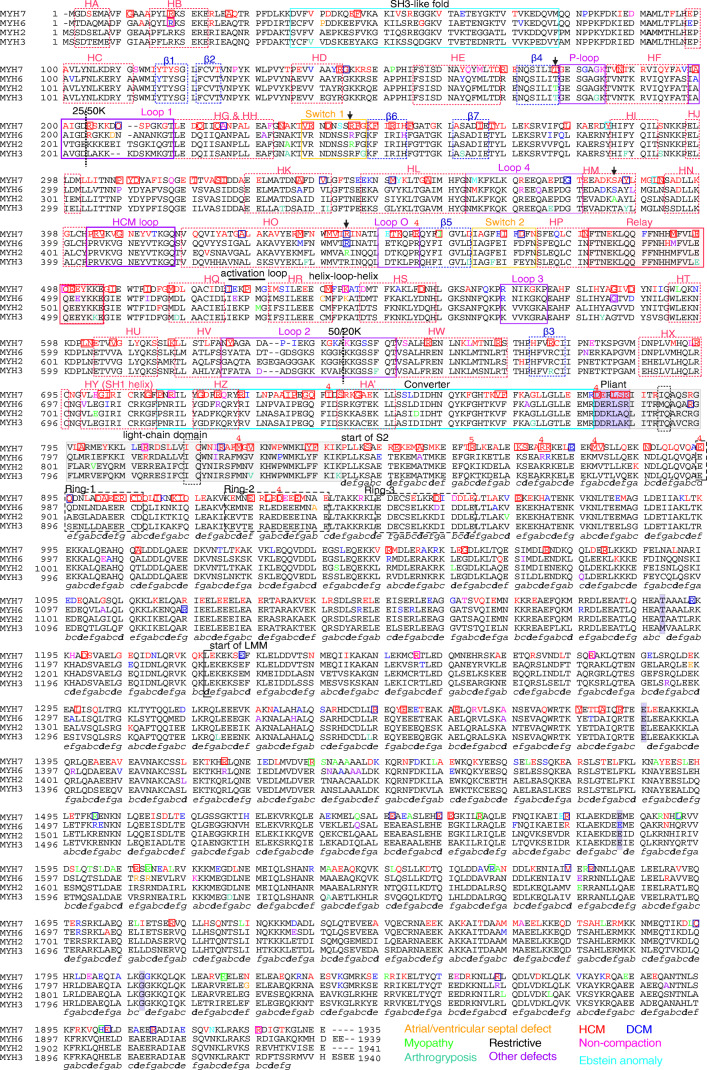
Positions of mutations each of the four myosin heavy chains. MYH7—β-cardiac myosin heavy chain, MYH6—α-cardiac myosin heavy chain, MYH3—embryonic myosin heavy chain and MYH2—MyHC2A. (See Supplemental Tables 1-4 for a list of the mutations and associated references). In subfragment-1 (S1), functional regions of the motor and lever are indicated. In subfragment-2 (S2), the three ‘rings’ of acidic residues are indicated. The 4 skip residues in LMM are highlighted. The heptad repeat of the coiled-coil sequence is indicated below the amino acid residues. Diseases caused by mutations in specific residues are shown in coloured font, as indicated by the legend. One to 2 mutations in the same residue are indicated by a box around the residue. Boxes are coloured differently to the residue if a second mutation causes a different disease. Residues in which there are more than 3 mutations are indicated by the number above the residue. Downward arrows indicate residues in which there is a mutation in 3 out of the 4 sequences

The second most common disease arising from mutations in the MYH7 gene is dilated cardiomyopathy (DCM). Mutations in MYH7 account for about 4% of all DCM, and the analysis here shows that 14% of all the mutations in MYH7 cause dilated cardiomyopathy. However, in a few residues, a missense mutation has been associated with either HCM or DCM (Supplementary Table 1, Fig. 2). DCM is caused by as many as 32 different genes, and while DCM was thought to be less common than HCM, it has recently been estimated to affect as many as 1 in 250 individuals (Hershberger et al. 2013).

About 5% of mutations are associated with left ventricular non-compaction (LVNC) (Fig. 1b, Supplementary Table 1). Reports of this disease are becoming more common (Towbin et al. 2015). In the embryonic heart, the myocytes are relatively loosely arranged into a spongy myocardium. During development, these cells become more organised and compacted. However, in LVNC, this compaction is incomplete, and the myocardium tends to contain multiple trabeculae as a result, affecting blood flow through the left ventricular chamber of the heart. LVNC can be associated with Ebstein’s anomaly (Attenhofer Jost et al. 2007), which involves a malformation of the tricuspid valve and an altered right ventricle, as well as defects in the septum, separating the left and right ventricle.

About 4% of mutations apparently have little effect on the heart, but mainly cause skeletal muscle myopathies such as Laing distal myopathy (LDM), myosin storage myopathy (MSM), hyaline body myopathy and multi-minicore disease, collectively known as myosinopathies (Tajsharghi and Oldfors 2013). Most of these mutations are found in a specific region of the coiled-coil tail (Colegrave and Peckham 2014), Supplementary Table 1), and commonly involve a mutation to a proline residue, or a deletion of a single amino acid, which likely disrupts the coiled coil (Parker et al. 2018). The intriguing question still remains as to why these mutations are not always associated with a cardiac phenotype.

An analysis of the positions of these mutations against their amino acid location shows that the ‘hot spots’ or peaks where mutations are more common are similar to those found in earlier studies with fewer mutations (Buvoli et al. 2008; Colegrave and Peckham 2014). These hot spots tend to be in key functional areas (Figs. 1a and 2, Supplemental Table 1). For example, 14 missense mutations are found in loop 1, and thus expected to affect the ATPase cycle. Mutations in loops 3, 4 and 2, the activation loop, the HCM loop and the helix-loop-helix motif, all proposed to enable the interaction of myosin with actin, account for 5% of all the mutations (Supplementary Table 1). There are 21 mutations in the relay helix, and 56 in the converter accounting for 8% of the total number of mutations. The pliant region, which is just 6 residues long, is highly mutated, with 13 mutations reported for this region. This sequence immediately follows the converter domain, providing an important connection between it and the light chain–binding domain (LCD), which forms the majority of the lever, and itself has over 30 mutations. The majority of the missense mutations in the myosin head are thus likely to affect force output by altering the ATPase cycle, the ability of myosin to interact with actin, or by transmission of force or movement by the lever.

Fifteen percent of the missense mutations in β-cardiac myosin heavy chain are found in subfragment-2 (S2) (Supplementary Table 1). Of the 191 mutations in S2, almost 60% are found in the first 120 residues, which contains three ‘rings’ of acidic residues (Fig. 2), interspersed by regions of basic residues. The myosin head has been shown to interact with the first ring of charge in its shutdown state, and mutations in the first charged ring may destabilise the formation of the IHM (Alamo et al. 2017). This interaction has been suggested to be mediated by loop 2 and the HCM loop, and thus, mutations in these loops may also affect the ability of myosin to form the shutdown state. Mutations in the second charged ring are likely to affect the ability of cMyBPC (cardiac myosin-binding protein C) to bind to myosin as discussed earlier (Colegrave and Peckham 2014), which would be expected to affect the ability of cMyBPC to modulate the contractile output of the heart.

The remaining 26% of mutations are found in LMM (Fig. 1a, Supplementary Table 1). Fifty-six percent cause HCM, 21% DCM, 14% cause skeletal muscle myopathy and 7% cause LVNC. Interestingly, while only 20% of the myosin mutations that cause HCM are found in LMM, this rises to 27% for LVNC, 38% for DCM and 90% for myopathy. The myopathy causing mutations are mostly found towards the distal end of LMM from residues 1430 onwards. Overall, the pattern of mutations in this region is again similar to that described earlier (Colegrave and Peckham 2014).

## Mutations in α-cardiac, embryonic and MyHC2A myosin heavy chain and disease

Mutations in α-cardiac myosin heavy chain are almost 10-fold less common than those in β-cardiac myosin heavy chain (Fig. 1c, Supplementary Table 2), with only 128 missense mutations described in HGMD. These mutations result in a wide range of cardiac diseases (Fig. 1d), of which DCM is the most common outcome (24%). The mutations occur throughout the sequence, with 45% occurring in the myosin head (S1), 19% in S2 and 35% in LMM. Thus, mutations in LMM are relatively more common in α-cardiac than in β-cardiac myosin heavy chain. With a lower number of mutations, a pattern is harder to spot, but mutations are relatively common in loop 1 and in the region of (but not in) the HCM loop. Interestingly, more mutations seem to occur in the distal region of S2 compared with the proximal region, in contrast to mutations in S2 in β-cardiac myosin heavy chain.

Mutations in the two skeletal myosin specific isoforms are still relatively uncommon, with 26 in MyHC2a and 34 in embryonic myosin heavy chain (Supplementary Tables 3 & 4, sourced from HGMD). Most of the mutations in embryonic myosin heavy chain (76%) are found in the S1, and interestingly, very few are found in S2 (Fig. 1e). Mutations in this myosin heavy chain cause Freeman-Sheldon (FSS) and Sheldon-Hall syndromes (Toydemir et al. 2006), both forms of arthrogryposis (severe multiple congenital contractures). These two syndromes are two of 10 different types of arthrogryposes and both strongly affect orofacial muscles. Three FSS mutations found in the motor domain resulted in a decreased rate of ATP hydrolysis, and of binding of ATP to myosin. This would be expected to reduce the rate at which the myosin head can detach from actin, and then increase the time in which the myosin remains detached from actin (Walklate et al. 2016). Embryonic myosin plays a key role in early muscle development, and its ablation in mice results in scoliosis (Agarwal et al. 2020), a phenotype also shown by Freeman-Sheldon syndrome patients, as well as numerous other defects.

Mutations in MyHC2A have only been relatively recently reported, with the first mutation described in 2000 (Martinsson et al. 2000). These mutations typically cause a variety of myopathies (Supplementary Table 4, sourced from HGMD), often associated with external ophthalmoplegia (weakness of the eye muscles), and type 2A muscle fibres are small or lacking. The distribution of missense mutations in MyHC2A is similar to that of the other myosins, with 58% in S1, 20% in S2 and 23% in LMM (Fig. 1e). To date, very little is known about the effects of these mutations on function.

## Discussion

The aim of this review was to briefly update our earlier analysis of mutations in β-cardiac myosin heavy chain (Colegrave and Peckham 2014) and extend it to three further myosin heavy chains in which mutations have begun to be reported. Despite the doubling in numbers of mutations described for β-cardiac myosin heavy chain in just 6 years, the overall pattern of mutations is somewhat similar to that described earlier. A similar pattern seems to be emerging for α-cardiac myosin heavy chain, with perhaps a slightly higher frequency of mutations in the filament forming region of this myosin. We still have much to learn about how these mutations exert their effects and how the expression of other myosin isoforms might change and compensate for or contribute to the overall phenotype of these disease mutations.

## Reflection on my interactions with Cris dos Remedios

I could not write this brief review without thinking back on my interactions with Cris over my career. I first remember meeting Cris when I was a PhD student, working with Roger Woledge in the Department of Physiology at University College London on muscle energetics. Roger was due to go to the International Union of Physiological Sciences meeting in Sydney, Australia, in 1983. Unfortunately, he was unable to go and sent me instead! It was the first time I had ever flown, and it was a harsh introduction to the effects of jet-lag. I proudly presented my poster, and in an oral discussion session, Cris (who was chairing) asked me to briefly discuss my findings with the audience. He then very kindly invited me back to his house for a party he was holding for the muscle research community.

**Fig. 3:**
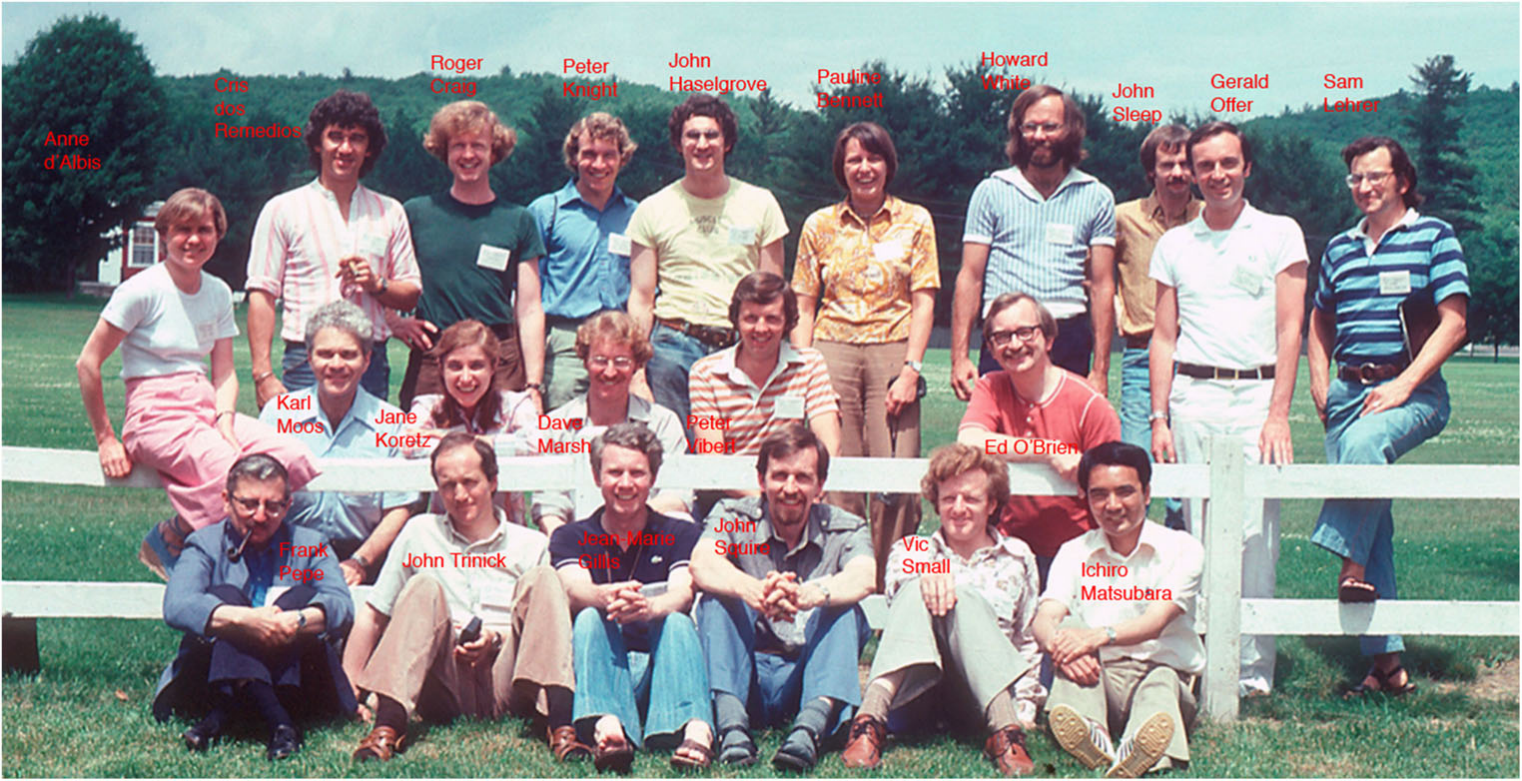
Cris dos Remedios and colleagues at a Gordon Conference

I have since met up with Cris on multiple occasions, and most recently we ran a session together in Edinburgh at the Joint 19th International Union of Pure and Applied Biophysics (IUPAB) and 11th European Biophysical Societies’ Association (EBSA) Congress in Edinburgh, Scotland, in July 2017. Cris was as enthusiastic as when I first met him all those years ago. He has been a major contributor to the field of muscle research (Fig. 3), and his contribution towards setting up the Sydney Heart Bank was a major achievement towards understanding cardiac disease.

## Electronic supplementary material

ESM 1 (PDF 741 kb)

## Abbreviations

FHC: Familial hypertrophic cardiomyopathy
HCM: Hypertrophic cardiomyopathy
DCM: Dilated cardiomyopathy
LDM: Laing distal myopathy
MSM: Myosin storage myopathy
S1: Subfragment-1 (myosin motor domain)
S2: Subfragment-2
LMM: Light meromyosin (myosin tail)
RLC: Regulatory light chain
ELC: Essential light chain
