# Supplementary material for: Disease mutations in striated muscle myosins
Source: Biophys Rev. 2020 Jul 10;12(4):887–94. doi: 10.1007/s12551-020-00721-5 (PMC7429545; doi:10.1007/s12551-020-00721-5)
Supplement: Supplementary file 1 — (PDF 741 kb) [file 12551_2020_721_MOESM1_ESM.pdf]

Supplemental Table 1: Missense mutations in MYH7 (β-cardiac myosin heavy chain) and their associated disease. HCM: hypertrophic cardiomyopathy. DCM: dilated cardiomyopathy. LVNC: Left Ventricular non-compaction.

Mutations	Myosin region	Disease	Reference
Ser4Leu		HCM	[1]
Gly10Ala		HCM	[2]
Gly10Arg		HCM	[3]
Ala13Thr		HCM	[4]
Arg17Cys		HCM	[5]
Arg17His		HCM	[6]
Ala23Trp		LVNC	[7]
Ala26Gly		HCM	[8]
Ala26Val		HCM	[9]
Gln27Arg		HCM	[3]
Val39Met	SH3-like fold	HCM	[10]
Asp41Asn	SH3-like fold	HCM	[11]
Gln44Term	SH3-like fold	LVNC	[12]
Glu45Asp	SH3-like fold	HCM	[3]
Phe46Cys	SH3-like fold	HCM	[3]
Phe46Ile	SH3-like fold	HCM	[13]
Val52Met	SH3-like fold	HCM	[14]
Arg54Gln	SH3-like fold	HCM	[3]
Arg54Term	SH3-like fold	HCM	[15]
Val59Ile	SH3-like fold	HCM	[8]
Glu62Lys	SH3-like fold	DCM	[3]
Thr70Ser	SH3-like fold	HCM	[16]
Pro81Ser		DCM	[17]
Ile87Thr		HCM	[14]
Asp89Gly		LVNC	[18]
Phe95Ile		HCM	[19]
Glu98Val		HCM	[3]
Ala100Thr		HCM	[20]
Ser111Phe		LVNC	[21]
Ile114Thr		HCM	[22]
Tyr115His		HCM	[23]
Tyr115Term		HCM	[6]
Thr116Ser		HCM	[13]
Tyr117Phe		HCM	[24]
Ser118Leu		HCM	[25]
Thr124Ile		HCM	[26]
Pro127Thr		LVNC	[27]
Thr135Ile		HCM	[19]
Glu137Lys		HCM	[22]
Val139Leu		DCM	[11]
Tyr142His		DCM	[17]
Arg143Gly		HCM	[28]
Arg143Trp		HCM	[29]
Arg143Gln		HCM	[30]
Gly144Asp		HCM	[24]
Gly144Val		DCM	[31]

Lys146Asn		HCM	[32]
Arg147Ser		HCM	[33]
Ser148Ile		HCM	[28]
Pro151Leu		Myopathy	[34]
Tyr162Cys		HCM	[26]
Tyr162His		HCM	[35]
Gln163Pro		LVNC	[36]
Met165Ile		LVNC	[18]
Thr167Ile		HCM	[3]
Asp168Asn		HCM	[24]
Arg169Gly		HCM	[37]
Arg169Lys		HCM	[5]
Arg169Ser		HCM	[5]
Glu170Lys		HCM	[38]
Thr177Ile		HCM	[39]
Thr177Ser		HCM	[40]
Gly178Arg	P-loop	HCM and SM	[17]
Gly181Arg	P-loop	DCM	[3]
Gly183Arg	P-loop	CM	[18]
Lys184Gln	P-loop	LVNC	[41]
Val186Leu		HCM	[32]
Asn187Lys		HCM	[26]
Asn187Ser		HCM	[24]
Thr188Asn		HCM	[10]
Arg190Thr		HCM	[42]
Gln193Arg		HCM	[3]
Tyr194Ser		HCM	[43]
Ala196Thr		HCM	[44]
Val197Ile		DCM	[3]
Ala199Thr	Loop-1	HCM	[14]
Ala199Val	Loop-1	CM	[37]
Ala200Thr	Loop-1	HCM	[45]
Ile201Thr	Loop-1	DCM	[46]
Arg204Cys	Loop-1	HCM	[24]
Arg204His	Loop-1	HCM	[10]
Arg204Leu	Loop-1	HCM	[3]
Ser205Cys	Loop-1	HCM	[47]
Lys207Gln	Loop-1	HCM	[28]
Gln209Glu	Loop-1	HCM	[47]
Gln209Lys	Loop-1	DCM	[48]
Ser210Arg	Loop-1	HCM	[25]
Pro211Leu	Loop-1	HCM	[28]
Gly214Asp	Loop-1	HCM	[49]
Leu216Val		HCM	[49]
Asp218Tyr		HCM	[19]
Gln219Leu		HCM	[50]
Gln219Glu		HCM	[50]
Gln222Lys		HCM	[26]
Gln222Arg		HCM	[38]
Ala223Thr		DCM	[51]
Ala223Val		LVNC	[7]

Asn224Ile		HCM	[47]
Ala226Thr		HCM	[52]
Leu227Val		HCM	[53]
Phe230Ser		LVNC	[18]
Asn232His		HCM	[3]
Asn232Ser		HCM	[54]
Ala233Ser		HCM	[55]
Thr235Asn		HCM	[19]
Val236Ala	Switch-1	HCM	[3]
Val236Ile	Switch-1	DCM	[56]
Arg237Gln	Switch-1	HCM	[3]
Arg237Trp	Switch-1	DCM	[57]
Asp239Asn	Switch-1	HCM	[43]
Asp239Glu	Switch-1	HCM	[24]
Ser241Phe	Switch-1	Ebstein anomaly	[58]
Arg243Cys	Switch-1	HCM	[59]
Arg243His	Switch-1	HCM	[60]
Phe244Leu	Switch-1	HCM	[26]
Phe244Cys	Switch-1	HCM	[3]
Gly245Glu	Switch-1	DCM	[61]
Lys246Gln		HCM	Rottbauer, 1996 HGMD
Lys246Ile		HCM	[3]
Phe247Leu		HCM	[1]
Ile248Thr		HCM	[5]
Ile248Phe		DCM	[61]
Arg249Gln		HCM	[62]
Arg249Gly		LVNC	[63]
Arg249Term		LVNC	[21]
Ile250Val		HCM	[3]
His251Asn		HCM	[64]
Phe252Cys		HCM	[65]
Phe252Leu		LVNC	[65]
Phe252Ser		HCM	[66]
Gly256Glu		HCM	[67]
Ala259Glu		HCM	[49]
Ile263Thr		HCM	[68]
Ile263Met		HCM	[23]
Thr265Asn		HCM	[24]
Thr265Ser		HCM	[3]
Tyr266Cys		HCM	[69]
Tyr266Term		LVNC	[18]
Leu267Val		HCM	[38]
Lys270Arg		HCM	[70]
Leu277Pro		HCM	[3]
Leu277Val		CM	[71]
Ala279Thr		DCM	[61]
Arg281Thr		LVNC	[72]
Tyr283Asp		Ebstein anomaly	[73]
Tyr283Cys		HCM	[5]
Tyr283His		LVNC	[74]
Ser291Phe		HCM	[75]

Leu301Gln		CM, non-compaction	[76]
Leu302Met		HCM	[6]
Ile303Met		HCM	[24]
Pro307Arg		HCM	[40]
Pro307His		HCM	[16]
Pro307Leu		HCM	[3]
Tyr308Cys		HCM	[25]
Tyr308Asn		HCM	[25]
Asp309Asn		HCM	[38]
Asp309Gly		HCM	Sabater-Molina, 2013 HGMD
Phe312Cys		HCM	[23]
Phe312Val		CM	[71]
Ile313Phe		HCM	[77]
Gln315Arg		LVNC	[19]
Glu317Gly		HCM	[16]
Thr318Asn		HCM	[24]
Thr318Pro		HCM	[77]
Val320Met		HCM	[78]
Ala321Val		HCM	[45]
Ser322Phe		HCM	[79]
Ser322Thr		HCM	[3]
Ile323Asn		HCM	[80]
Ile323Thr		HCM	[3]
Ala326Pro		HCM	[81]
Glu328Gly		HCM	[53]
Ala335Ser		HCM	[3]
Ala335Thr		HCM	[82]
Val338Met		HCM	[83]
Val338Ala		DCM	[56]
Phe341Leu		HCM	[6]
Glu344Lys		DCM	[84]
Glu345Asp		DCM	[31]
Lys346Glu		DCM	[31]
Met349Thr		HCM	[85]
Met349Val		DCM	[3]
Tyr350Asn		Ebstein anomaly	[73]
Lys351Glu		HCM	[28]
Glu354Ser		HCM	[5]
Ala355Ser		HCM	[3]
Ala355Thr		HCM	[10]
Ala355Val		HCM	[38]
Met362Arg	Loop 4	Ebstein anomaly, LVNC	[86]
Arg369Gln	Loop 4	CM, non-compaction	[87]
Glu374Val	Loop 4	HCM	[3]
Gly377Arg	Loop 4	DCM	[88]
Gly377Ser	Loop 4	DCM	[69]
Thr378Pro	Loop 4	HCM	[3]
Glu379Lys		HCM	[38]
Ala381Asp		HCM	[3]
Asp382Tyr		HCM	[64]
Lys383Arg		HCM	[3]

Lys383Asn		HCM	[89]
Ser384Pro		HCM	[19]
Ala385Val		HCM	[23]
Tyr386His		HCM	[50]
Tyr386Cys		DCM	[90]
Leu387Phe		CM	[91]
Met388Thr		HCM	[92]
Gly389Glu		HCM	[3]
Leu390Pro		Ebstein anomaly	[73]
Leu390Val		HCM	[78]
Asn391Thr		HCM	[13]
Ala393Val		HCM	[11]
Asp394Glu		HCM	[38]
Lys397Glu		LVNC	Mokhatar, 2017 HGMD
Gly398Glu		HCM	[70]
Pro402Ser	HCM Loop	HCM	[24]
Arg403Gln	HCM Loop	HCM	[93]
Arg403Gly	HCM Loop	HCM	[3]
Arg403Leu	HCM Loop	HCM	[94]
Arg403Pro	HCM Loop	LVNC	[18]
Arg403Trp	HCM Loop	HCM	[94]
Val404Leu	HCM Loop	HCM	[44]
Val404Met	HCM Loop	HCM	[23]
Val406Met	HCM Loop	HCM	[95]
Gly407Cys	HCM Loop	HCM	[96]
Gly407Val	HCM Loop	HCM	[23]
Asn408Lys	HCM Loop	HCM	[14]
Tyr410Asp	HCM Loop	HCM	[3]
Val411Ile	HCM Loop	HCM	[29]
Thr412Asn	HCM Loop	DCM	[46]
Gln418Lys		HCM	[47]
Tyr422Cys		CM	[97]
Ala423Val		HCM	[3]
Gly425Arg		HCM	[98]
Ala426Thr		HCM	[19]
Ala426Val		DCM	[84]
Leu427Met		HCM	[99]
Ala428Asp		LVNC	[100]
Ala428Val		HCM	[10]
Ala430Glu		HCM	[101]
Val431Leu		HCM	[24]
Arg434Thr		HCM	[19]
Met435Arg		HCM	[24]
Met435Thr		HCM	[53]
Asn437Asp		HCM	[13]
Trp438Term		MSM	[102]
Met439Arg		LVNC	[103]
Met439Leu		HCM	[3]
Met439Thr		HCM	[19]
Val440Ala		HCM	[14]
Val440Met		HCM and SM	[23]

Thr441Met		CM and distal myopathy	[104]
Arg442Cys		HCM	[25]
Arg442His		DCM	[105]
Ile443Thr		HCM	[10]
Ile443Val		HCM	[10]
Asn444Ser		HCM	[106]
Thr446Pro		HCM	[107]
Glu448Lys		LVNC	[7]
Thr449Asn		HCM	[82]
Thr449Ile		HCM	[3]
Thr449Ser		HCM	[3]
Lys450Glu		HCM	[108]
Lys450Thr		HCM	[98]
Gln451Pro		HCM	[3]
Arg453Cys		HCM	[109]
Arg453His		HCM	[53]
Arg453Ser		HCM	[110]
Arg453Leu		HCM	Haluza, 2000 HGMD
Arg453Pro		HCM	[82]
Ile457Arg		Myopathy with cardiac involvement	[111]
Ile457Thr		HCM	[17]
Asp461Glu		HCM	[19]
Ala463Asp	Switch 2	HCM	[82]
Glu466Gln	Switch 2	DCM	[84]
Ile467Leu	Switch 2	HCM	[112]
Ile467Thr	Switch 2	HCM	[17]
Phe468Leu	Switch 2	DCM	[90]
Asp469Asn	Switch 2	HCM	[24]
Asp469Tyr	Switch 2	DCM	[90]
Asn471Ser	Switch 2	HCM	[38]
Gln475His	Relay	DCM	[17]
Gln475Lys	Relay	DCM	[17]
Leu476Val	Relay	HCM	[3]
Ile478Asn	Relay	HCM	[6]
Asn479Thr	Relay	HCM	[11]
Asn479Ser	Relay	HCM	[10]
Glu483Cys	Relay	HCM	[25]
Glu483Lys	Relay	HCM	[113]
Phe489Ile	Relay	DCM	[17]
Met493Leu	Relay	HCM	[59]
Met493Ile	Relay	HCM	[114]
Met493Lys	Relay	HCM	[115]
Met493Val	Relay	HCM	[116]
Glu497Asp	Relay	HCM	[60]
Glu497Gly	Relay	HCM	[5]
Gln498Arg	Relay	HCM	[38]
Gln498Glu	Relay	LVNC	[117]
Glu499Gly	Relay	HCM	[3]
Glu499Lys	Relay	HCM	[118]
Glu500Ala	Relay	HCM	[28]

Tyr501Cys	Relay	HCM	[119]
GLy505Val		HCM	[3]
Ile506Phe		HCM	[25]
Ile506Thr		HCM	[19]
Ile511Phe		HCM	[98]
Ile511Thr		HCM	[23]
Phe513Cys		HCM	[120]
Gly514Asp		HCM	[3]
Met515Thr		HCM	[69]
Met515Val		HCM	[121]
Met515Arg		HCM	[23]
Asp516Glu		HCM	[122]
Leu517Met		HCM	[123]
Ile524Val	Activation loop	DCM	[61]
Glu525Lys	Activation loop	DCM	[90]
Pro527Leu	Activation loop	HCM	[40]
Pro527Thr	Activation loop	HCM	[3]
Gly529Asp	Activation loop	DCM	[17]
Ile530Val	Helix-loop-helix	HCM	[124]
Met531Arg	Helix-loop-helix	HCM	[125]
Ser532Pro	Helix-loop-helix	DCM	[105]
Ile533Val	Helix-loop-helix	DCM	[61]
Glu536Asp	Helix-loop-helix	HCM	[5]
Met539Ile	Helix-loop-helix	HCM	[126]
Met539Val	Helix-loop-helix	HCM	[127]
Met539Leu	Helix-loop-helix	HCM	[43]
Phe540Leu	Helix-loop-helix	DCM	[128]
Lys542Arg	Helix-loop-helix	HCM	[20]
Lys542Thr	Helix-loop-helix	LVNC	[129]
Ala543Thr	Helix-loop-helix	DCM	[17]
Thr544Ala	Helix-loop-helix	DCM	[3]
Asp545Asn	Helix-loop-helix	CM, non-compaction	[76]
Ala550Val	Helix-loop-helix	DCM	[46]
Asp554Glu	Helix-loop-helix	HCM	[25]
Asp554Tyr	Helix-loop-helix	HCM	[130]
Ala561Thr		HCM	[17]
Gln564Glu		DCM	[17]
Arg567His	Loop-3	DCM	[61]
Gly571Arg	Loop-3	HCM	[24]
His576Arg		HCM	[119]
His581Arg		HCM	[131]
Ala583Val		HCM	[1]
Gly584Arg		HCM	[109]
Gly584Ser		HCM	[29]
Ile585Phe		HCM	[3]
Val586Ala		HCM	[5]
Asp587Asn		HCM	[3]
Asp587Val		HCM	[132]
Ile590Val		HCM	[107]
Ile591Thr		DCM	[17]
Leu594Met		Myopathy	[133]

Asn597Lys		DCM	[61]
Leu601Val		HCM	[78]
Leu601Phe		HCM	[55]
Asn602Ser		HCM	[132]
Asn602Tyr		HCM	[3]
Glu603Lys		HCM	[24]
Val606Leu		HCM	[132]
Val606Met		HCM	[109]
GLy607Asp		HCM	[16]
Tyr609Cys		HCM	[17]
Ser612Pro		HCM	[25]
Lys615Gln		HCM	[98]
Lys615Asn		HCM	[134]
Thr619Ile		HCM	[3]
Leu620Pro		HCM	[135]
Tyr624Asn	Loop 2	HCM	[136]
Tyr624Cys	Loop 2	HCM	[16]
Pro630Ser	Loop 2	DCM	[3]
Ile631Thr	Loop 2	DCM	[3]
Lys635Term	Loop 2	DCM	[17]
Gly636Ser	Loop 2	HCM	[24]
Lys637Glu	Loop 2	DCM	[137]
Lys639Glu	Loop 2	LVNC	[18]
Ser642Leu	Loop 2	DCM	[51]
Ser648Leu		LVNC	[36]
Arg652Gly		HCM	[138]
Arg652Lys		HCM	[22]
Arg652Thr		HCM	[96]
Lys657Gln		HCM	[139]
Leu658Val		LVNC	[140]
Met659Ile		HCM	[10]
Met659Thr		DCM	[141]
Arg660Asn		HCM	[112]
Arg663Cys		HCM	[23]
Arg663Ser		HCM	[10]
Arg663His		HCM	[142]
His668Asn		HCM	[11]
Val670Ala		Myaglia	[143]
Arg671Cys		HCM	[10]
Arg671His		HCM	[19]
Cys672Phe		DCM	[3]
Glu677Val		LVNC	[7]
Met690Thr		HCM	[16]
Leu693Arg		LVNC	[7]
Arg694Cys		HCM	[29]
Arg694His		HCM	[144]
Arg694Leu		HCM	[145]
Asn696Ser		HCM	[146]
Val698Ala		HCM	[32]
Glu700Gly		LVNC	[36]
Gly701Asp		HCM	[147]

Gly701Ser		HCM	[3]
Ile702Asn		HCM	[5]
Ile702Val		HCM	[114]
Arg703His		HCM	[25]
Cys705Trp		HCM	[17]
Gly708Ala		HCM	[80]
Gly708Asp		HCM	[148]
Pro710Arg	Converter	HCM	[64]
Pro710His	Converter	HCM	[149]
Pro710Leu	Converter	HCM	[5]
Arg712His	Converter	LVNC	[7]
Arg712Leu	Converter	HCM	[150]
Gly716Arg	Converter	HCM	[120]
Gly716Ala	Converter	HCM	[38]
Asp717Gly	Converter	HCM	[151]
Arg719Trp	Converter	HCM	[120]
Arg719Gln	Converter	HCM	[152]
Arg719Leu	Converter	HCM	[14]
Arg719Pro	Converter	HCM	[1]
Gln720His	Converter	LVNC	[74]
Arg721Lys	Converter	HCM	[69]
Arg723His	Converter	HCM	[43]
Arg723Cys	Converter	HCM	[109]
Arg723Gly	Converter	HCM	[153]
Ile724Asn	Converter	DCM	[61]
Arg726Lys	Converter	HCM	[24]
Ala728Val	Converter	HCM	[154]
Ala729Pro	Converter	HCM	[4]
Ile730Asn	Converter	HCM	[155]
Ile730Met	Converter	HCM	[24]
Ile730Thr	Converter	HCM	[47]
Pro731Ala	Converter	HCM	[5]
Pro731Leu	Converter	HCM	[156]
Pro731Ser	Converter	HCM	[25]
Gly733Arg	Converter	DCM	[17]
Gly733Glu	Converter	HCM	[10]
Gly733Val	Converter	HCM	[25]
Gln734Glu	Converter	HCM	[123]
Gln734Pro	Converter	HCM	[98]
Ile736Leu	Converter	HCM	[50]
Ile736Val	Converter	HCM	[14]
Ile736Thr	Converter	HCM	[29]
Ile736Met	Converter	HCM	[8]
Ser738Asn	Converter	HCM	[157]
Ser738Thr	Converter	HCM	[24]
Arg739Ser	Converter	HCM	[5]
Lys740Asn	Converter	HCM	[19]
Gly741Ala	Converter	HCM	[23]
Gly741Arg	Converter	HCM	[67]
Gly741Trp	Converter	HCM	[8]
Ala742Glu	Converter	HCM	[158]

Glu743Asp	Converter	HCM	[159]
Ser748Pro	Converter	DCM	[160]
Leu749Gln	Converter	DCM	[56]
Phe758Cys	Converter	HCM	[3]
Thr761Asn	Converter	HCM	[161]
Lys762Arg	Converter	HCM	[5]
Val763Met	Converter	HCM	[162]
Val763Gly	Converter	HCM	[28]
Phe764Tyr	Converter	HCM	[114]
Phe764Leu	Converter	DCM	[105]
Lys766Gln	Converter	HCM	[5]
Gly768Arg	Converter	HCM	[43]
Leu769Pro	Converter	HCM	[24]
Glu774Val	Converter	HCM	[163]
Arg777Lys	Converter	DCM	[84]
Asp778Asn	Pliant	HCM	[97]
Asp778Gly	Pliant	HCM	[164]
Asp778Val	Pliant	HCM	[23]
Asp778Glu	Pliant	HCM	[122]
Glu779Asp	Pliant	HCM	[50]
Glu779Term	Pliant	HCM	[163]
Leu781Met	Pliant	HCM	[19]
Leu781Pro	Pliant	HCM	[5]
Ser782Arg	Pliant	HCM	[70]
Ser782Asn	Pliant	HCM	[26]
Arg783Cys	Pliant	HCM	[24]
Arg783His	Pliant	HCM	[25]
Arg783Pro	Pliant	CM and distal myopathy	[158]
Ile785Val	LCD	HCM	[3]
Arg787Cys	LCD	HCM	[162]
Arg787His	LCD	HCM	[10]
Arg793Gln	LCD	HCM	[17]
Leu796Phe	LCD	HCM	[29]
Ala797Pro	LCD	HCM	[92]
Ala797Thr	LCD	HCM	[165]
Tyr801Asn	LCD	HCM	[3]
Leu805Pro	LCD	DCM	[84]
Arg807Gly	LCD	LVNC	[27]
Arg807His	LCD	HCM	[77]
Asp809Tyr	LCD	HCM	[3]
Leu811Pro	LCD	HCM	[5]
Ile814Ser	LCD	DCM	[3]
Gln815Pro	LCD	HCM	[126]
Asn817Lys	LCD	HCM	[24]
Ile818Asn	LCD	CM, LVNC	[140]
Arg819Gln	LCD	HCM	[38]
Arg819Trp	LCD	DCM	[3]
Ala820Asp	LCD	HCM	[166]
Phe821Ser	LCD	HCM	[3]
Met822Leu	LCD	HCM	[45]
Met822Val	LCD	HCM	[1]

Met822Thr	LCD	HCM	[98]
Gly823Glu	LCD	HCM	[98]
Val824Ala	LCD	HCM	[5]
Val824Leu	LCD	HCM	[3]
Val824Ile	LCD	HCM	[29]
Trp827Cys	LCD	HCM	[50]
Pro828Ser	LCD	HCM	[20]
Tyr833Cys	LCD	HCM	[3]
Tyr833His	LCD	HCM	[24]
Phe834Leu	LCD	HCM	[5]
Phe834Tyr	LCD	HCM	[3]
Lys835Thr	LCD	HCM	[4]
Ile836Met	LCD	HCM	[24]
Ile836Thr	LCD	HCM	[147]
Pro838Leu	S2	HCM	[167]
Leu840Met	S2	HCM	[168]
Ser842Arg	S2	HCM	[169]
Ser842Asn	S2	HCM	[5]
Ser842Gly	S2	HCM	[24]
Glu844Lys	S2	HCM	[122]
Arg845Gly	S2	HCM	[170]
Glu846Gln	S2	HCM	[78]
Glu846Lys	S2	HCM	[24]
Lys847Glu	S2	HCM	[64]
Glu848Gly	S2	HCM	[5]
Met849Thr	S2	HCM	[171]
Ala850Asp	S2	HCM	[172]
Ala850Thr	S2	HCM	[4]
Met852Thr	S2	HCM	[10]
Lys853Gln	S2	HCM	[19]
Thr857Ile	S2	HCM	[3]
Thr857Pro	S2	HCM	[173]
Arg858Cys	S2	HCM	[23]
Arg858His	S2	HCM	[98]
Arg858Gly	S2	HCM	[83]
Arg858Pro	S2	HCM	[55]
Arg858Ser	S2	HCM	[131]
Lys860Glu	S2	HCM	[77]
Glu861Term	S2	DCM	[17]
Ala862Val	S2	HCM	[47]
Lys865Arg	S2	HCM	[55]
Lys865Glu	S2	HCM	[24]
Lys865Met	S2	HCM	[14]
Ser866Tyr	S2	HCM	[170]
Ser866Pro	S2	HCM	[45]
Ala868Pro	S2	HCM	[50]
Arg869Cys	S2	HCM	[120]
Arg869Gly	S2	HCM	[174]
Arg869His	S2	HCM	[43]
Arg869Leu	S2	HCM	[3]
Arg870Cys	S2	HCM	[175]

Arg870His	S2	HCM	[176]
Arg870Leu	S2	HCM	[177]
Leu873Pro	S2	HCM	[114]
Glu874Gln	S2	DCM	[178]
Glu874Val	S2	DCM	[84]
Met877Ile	S2	HCM	[3]
Met877Lys	S2	HCM	[95]
Val878Ala	S2	HCM	[179]
Val878Met	S2	HCM	[19]
Val878Gly	S2	HCM	[50]
Gln882Glu	S2	HCM	[28]
Asn885Lys	S2	HCM	[114]
Asn885Thr	S2	HCM	[180]
Gln888Lys	S2	HCM	[135]
Leu889His	S2	HCM	[64]
Gln890Pro	S2	HCM	[6]
Gln892Lys	S2	HCM	[13]
Ala893Val	S2	DCM	[90]
Glu894Gln	S2	HCM	[14]
Glu894Lys	S2	HCM	[19]
Glu894Gly	S2	HCM	[23]
Glu894Asp	S2	DCM	[3]
Gln895Lys	S2	HCM	[3]
Gln895Glu	S2	DCM	[3]
Leu898Val	S2	HCM	[5]
Asp900Glu	S2	HCM	[14]
Asp900Gly	S2	HCM	[3]
Asp900Val	S2	HCM	[24]
Ala901Gly	S2	HCM	[119]
Ala901Pro	S2	HCM	[92]
Glu902Lys	S2	CM restrictive	[24]
Glu903Lys	S2	HCM	[23]
Glu903Gly	S2	HCM	[162]
Glu903Gln	S2	HCM	[16]
Arg904Cys	S2	DCM	[181]
Arg904His	S2	DCM	[17]
Cys905Arg	S2	CM, non-compaction	[182]
Cys905Phe	S2	HCM	[29]
Cys905Tyr	S2	HCM	[19]
Asp906Asn	S2	HCM	[3]
Asp906Gly	S2	HCM	[138]
Gln907Lys	S2	HCM	[3]
Leu908Val	S2	HCM	[67]
Ile909Met	S2	HCM	[70]
Ile909Val	S2	HCM	[3]
Lys910Gln	S2	DCM	[17]
Lys912Asn	S2	HCM	[183]
Lys912Gln	S2	HCM	[183]
Ile913Met	S2	HCM	[24]
Ile913Thr	S2	HCM	[19]
Gln914His	S2	HCM	[5]

Leu915Pro	S2	LVNC	[36]
Glu916Lys	S2	HCM	[3]
Glu921Lys	S2	HCM	[23]
Glu924Gln	S2	HCM	[29]
Glu924Lys	S2	HCM	[109]
Arg925Gly	S2	DCM	[184]
Leu926Pro	S2	HCM	[126]
Leu926Val	S2	HCM	[3]
Glu927Lys	S2	HCM	[53]
Asp928Ala	S2	HCM	[3]
Asp928Asn	S2	HCM	[29]
Asp928Gly	S2	HCM	[6]
Asp928Val	S2	HCM	[185]
Glu929Lys	S2	CM, LVNC	[63]
Glu930Lys	S2	HCM	[26]
Glu930Gln	S2	HCM	[55]
Glu931Ala	S2	HCM	[157]
Glu931Gly	S2	HCM	[6]
Glu931Lys	S2	HCM	[23]
Met932Lys	S2	HCM	[43]
Glu935Lys	S2	HCM	[25]
Glu935Val	S2	HCM	[186]
Arg941Cys	S2	LVNC	[7]
Glu949Lys	S2	HCM	[109]
Glu949Val	S2	HCM	[24]
Asp953His	S2	HCM	[23]
Asp953Val	S2	HCM	[23]
Ile954Val	S2	DCM	[24]
Asp955Asn	S2	CM, non-compaction	[76]
Asp956Asn	S2	HCM	[97]
Leu957Arg	S2	HCM	[183]
Glu958Lys	S2	HCM	[97]
Leu959Arg	S2	HCM	[183]
Leu961Arg	S2	HCM	[112]
Leu961Pro	S2	LVNC	[36]
Leu961Val	S2	HCM	[3]
Ala962Asp	S2	HCM	[183]
Val964Leu	S2	DCM	[57]
Glu965Gly	S2	HCM	[3]
Glu965Lys	S2	HCM	[70]
Glu967Lys	S2	HCM	[5]
His969Pro	S2	HCM	[3]
Ala970Val	S2	DCM	[57]
Glu981Lys	S2	DCM	[48]
Met982Thr	S2	HCM	[25]
Lys991Asn	S2	DCM	[61]
Leu992Met	S2	HCM	[16]
Lys994Arg	S2	HCM	[19]
Ala1006Thr	S2	HCM	[157]
Lys1016Glu	S2	HCM	[3]
Thr1019Asn	S2	DCM	[46]

Lys1022Glu	S2	HCM	[3]
Leu1027Pro	S2	DCM	[17]
Leu1038Pro	S2	DCM	[137]
Val1044Ala	S2	HCM	[50]
Arg1045Cys	S2	HCM	[70]
Arg1045His	S2	HCM	[25]
Arg1045Leu	S2	HCM	[5]
Met1046Ile	S2	HCM	[3]
Arg1050Gln	S2	HCM	[19]
Ala1051Ala	S2	HCM	[106]
Ala1051Val	S2	DCM	[90]
Arg1053Gln	S2	DCM	[187]
Glu1056Asp	S2	HCM	[38]
Gly1057Asp	S2	HCM	[32]
Gly1057Ser	S2	HCM	[23]
Glu1070Lys	S2	HCM	[3]
Asp1077Glu	S2	HCM	[3]
Arg1079Gln	S2	HCM	[188]
Arg1079Gln	S2	Sudden unexpected death	[189]
Leu1090Val	S2	HCM	[13]
Asn1091Asp	S2	Sudden cardiac death	[190]
Glu1095Gly	S2	DCM	[3]
Asp1096Tyr	S2	DCM	[57]
Glu1097Lys	S2	HCM	[3]
Gly1101Ser	S2	HCM	[112]
Lys1109Glu	S2	HCM	[3]
Arg1114His	S2	HCM	[3]
Glu1116Lys	S2	HCM	[50]
Glu1119Lys	S2	HCM	[19]
Glu1120Lys	S2	HCM	[14]
Glu1123Gln	S2	HCM	[186]
Glu1125Gln	S2	HCM	[3]
Ala1128Thr	S2	HCM	[24]
Glu1133Lys	S2	HCM	[14]
Leu1135Arg	S2	HCM	[10]
Arg1136His	S2	HCM	[24]
Glu1142Lys	S2	HCM	[19]
Leu1143Arg	S2	HCM	[3]
Glu1152Val	S2	DCM	[61]
Gly1155Glu	S2	DCM	[3]
Thr1157Ala	S2	LVNC	Mokhtar, 2017 HGMD
Val1159Met	S2	HCM	[3]
Gln1160Arg	S2	HCM	[25]
Glu1162Lys	S2	HCM	[3]
Lys1165Glu	S2	HCM	[3]
Glu1168Asp	S2	HCM	[13]
Leu1183Gln	S2	HCM	[3]
Arg1193His	S2	DCM	[90]
Arg1193Ser	S2	DCM	[46]
His1196Tyr	S2	LVNC	[27]
Asp1198Gly	S2	HCM	[6]

Asp1198Tyr	S2	HCM	[77]
Ser1199Arg	S2	HCM	[24]
Gly1204Arg	S2	HCM	[24]
Glu1205Lys	S2	HCM	[25]
Asp1208Asn	S2	HCM	[25]
Asp1208Gly	S2	HCM	[191]
Asn1209Ser	S2	DCM	[56]
Val1213Met	S2	HCM	[24]
Gln1215His	S2	HCM	[114]
Lys1216Met	S2	HCM	[107]
Glu1218Gln	LMM	HCM	[10]
Ser1222Gly	LMM	HCM	[3]
Glu1223Gln	LMM	DCM	[17]
Glu1223Lys	LMM	DCM	[84]
Asn1234Thr	LMM	HCM	[24]
Ile1239Val	LMM	CM, right ventricular	[192]
Lys1242Lys	LMM	Sudden cardiac death	[193]
Leu1245Gln	LMM	DCM	[3]
Arg1250Pro	LMM	HCM	[194]
Arg1250Trp	LMM	CM, non-compaction	[87]
Ala1263Glu	LMM	HCM	[50]
Arg1268His	LMM	Cardiac defects	[195]
Arg1268Pro	LMM	HCM	[3]
Leu1273Pro	LMM	HCM	[25]
Arg1277Gln	LMM	HCM	[13]
Arg1277Pro	LMM	LVNC	[27]
Glu1286Lys	LMM	DCM	[90]
Glu1293Lys	LMM	HCM	[82]
Leu1297Gln	LMM	HCM	[19]
Leu1297Val	LMM	HCM	[50]
Gln1300Leu	LMM	HCM	[3]
Arg1303Gly	LMM	DCM	[141]
Arg1303Term	LMM	DCM	[31]
Asp1314Glu	LMM	DCM	[196]
Ala1325Val	LMM	DCM	[3]
Asn1327Lys	LMM	HCM	[197]
Ala1328Thr	LMM	DCM	[61]
Ala1332Thr	LMM	DCM	[17]
Gln1334Term	LMM	HCM	[197]
Ser1335Leu	LMM	DCM	[3]
Arg1337Gln	LMM	HCM	[25]
Arg1344Gln	LMM	HCM	[198]
Arg1344Trp	LMM	DCM	[90]
Gln1346Term	LMM	DCM	[149]
Tyr1347Cys	LMM	HCM	[147]
Glu1348Gln	LMM	HCM	[19]
Glu1348Lys	LMM	HCM	[19]
Glu1350Lys	LMM	DCM	[199]
Thr1351Met	LMM	HCM	[55]
Glu1356Gln	LMM	HCM	Sabatar-Molina, 2013 HGMD
Glu1356Lys	LMM	HCM	[23]

Arg1359Cys	LMM	CM, non-compaction	[65]
Val1360Ile	LMM	HCM	[19]
Ala1364Pro	LMM	LVNC	[74]
Gln1370Lys	LMM	HCM	[3]
Tyr1375His	LMM	HCM	[24]
Tyr1375Cys	LMM	HCM	[5]
Tyr1375Term	LMM	LVNC	[18]
Thr1377Met	LMM	HCM	[25]
Asp1378His	LMM	HCM	[3]
Ala1379Asp	LMM	HCM	[3]
Ala1379Thr	LMM	HCM	[200]
Arg1382Trp	LMM	HCM	[10]
Arg1382Gln	LMM	HCM	[99]
Leu1386Phe	LMM	HCM	[3]
Glu1387Lys	LMM	HCM	[24]
Ala1400Gly	LMM	HCM	[194]
Ala1403Asp	LMM	HCM	[24]
Val1404Met	LMM	HCM	[19]
Leu1414Met	LMM	HCM	[162]
Thr1417Ile	LMM	HCM	[6]
Arg1420Gln	LMM	HCM	[201]
Arg1420Leu	LMM	HCM	[3]
Arg1420Trp	LMM	HCM	[23]
Glu1426Lys	LMM	DCM	[46]
Leu1428Ser	LMM	HCM	[124]
Arg1434Cys	LMM	HCM/DCM	[17]
Ala1434Pro	LMM	Myopathy	[202]
Ser1435Pro	LMM	Myopathy	[203]
Ala1437Pro	LMM	Myopathy	[202]
Ala1439Pro	LMM	Myopathy	[204]
Asp1443Asn	LMM	HCM	[24]
Lys1444Glu	LMM	DCM	[56]
Asn1448Ser	LMM	HCM	[3]
Asp1450Asn	LMM	DCM	[90]
Leu1453Pro	LMM	Myopathy	[205]
Ala1454Thr	LMM	HCM	[119]
Glu1455Term	LMM	HCM	[188]
Glu1455Lys	LMM	HCM	[206]
Lys1459Asn	LMM	HCM	[23]
Ser1465Leu	LMM	DCM	[17]
Leu1467Val	LMM	Myopathy with cardiac involvement	[207]
Glu1468Gln	LMM	HCM	[24]
Glu1468Lys	LMM	HCM	[24]
Ser1470Pro	LMM	DCM	[61]
Gln1471Term	LMM	DCM	[61]
Glu1473Gly	LMM	HCM	[3]
Arg1475Cys	LMM	HCM	[25]
THr1479Ile	LMM	HCM	[3]
Leu1481Pro	LMM	Myopathy	[208]
Ala1487Thr	LMM	Myopathy	[209]

Tyr1488Cys	LMM	CM, non-compaction	[140]
Glu1489Gly	LMM	HCM	[25]
Ser1491Cys	LMM	HCM	[197]
Leu1492Pro	LMM	Myopathy	[133]
His1494Leu	LMM	HCM	[25]
Glu1496Ala	LMM	HCM	[210]
Arg1500Trp	LMM	DCM	[187]
Arg1500Pro	LMM	Myopathy	[211]
Gln1506Term	LMM	DCM	[17]
Ile1509Leu	LMM	HCM	[3]
Asp1511Ala	LMM	HCM	[3]
Thr1513Ser	LMM	HCM	[23]
Glu1514Lys	LMM	DCM	[56]
His1524Arg	LMM	HCM	[114]
Arg1530Term	LMM	CM	[212]
Glu1536Lys	LMM	DCM	[17]
Glu1541Pro	LMM	Myopathy	[208]
Glu1546Gln	LMM	HCM	[3]
Glu1546Asp	LMM	DCM	[3]
Glu1548Val	LMM	DCM	[3]
Ala1549Pro	LMM	Myopathy	[213]
Glu1554Gln	LMM	DCM	[214]
Glu1554Lys	LMM	HCM	[3]
Glu1555Gly	LMM	HCM	[99]
Glu1555Lys	LMM	HCM	[215]
Arg1560Gln	LMM	HCM	[24]
Arg1560Pro	LMM	Myopathy	[216]
Gln1567Term	LMM	Myopathy	[217]
Glu1573Lys	LMM	Ebstein anomaly	[73]
Arg1574Gln	LMM	HCM	[218]
Arg1574Trp	LMM	DCM	[61]
Arg1588Pro	LMM	Myopathy	[207]
Asn1589Lys	LMM	HCM	[19]
Leu1591Gln	LMM	Sudden cardiac death	[219]
Leu1591Pro	LMM	Myopathy	[220]
Ser1596Leu	LMM	HCM	[3]
Leu1597Arg	LMM	Myopathy	[221]
Thr1599Pro	LMM	Myopathy	[208]
Leu1601Pro	LMM	Myopathy	[222]
Ala1603Pro	LMM	Myopathy	[220]
Arg1606Cys	LMM	HCM	[80]
Arg1606His	LMM	HCM	[114]
Arg1608Pro	LMM	Myopathy, DCM	[223]
Arg1608Ser	LMM	Myopathy	[222]
Glu1610Gln	LMM	DCM	[224]
Ala1611Val	LMM	HCM	[225]
Leu1612Pro	LMM	Myopathy	[208]
Glu1619Lys	LMM	DCM	[57]
Leu1622Phe	LMM	HCM	[24]
Arg1634Cys	LMM	DCM	[46]
Ala1636Pro	LMM	Myopathy	[208]

Ala1637Thr	LMM	HCM	[50]
Glu1638Lys	LMM	HCM	[13]
Gln1640Arg	LMM	HCM	[3]
Leu1646Pro	LMM	Myopathy	[208]
Gln1647His	LMM	HCM	[13]
Asp1652Tyr	LMM	HCM	[106]
Ala1660Glu	LMM	DCM	[17]
Arg1662His	LMM	DCM	[17]
Ala1663Pro	LMM	Myopathy	[211]
Asn1664Lys	LMM	HCM	[24]
Lys1668Glu	LMM	HCM	[3]
Val1674Met	LMM	HCM	[3]
Asn1674Leu	LMM	DCM	[3]
Arg1676Pro	LMM	Myopathy	[226]
Arg1677Cys	LMM	HCM	[3]
Arg1677His	LMM	DCM	[17]
Val1691Met	LMM	HCM	[10]
Glu1696Asp	LMM	HCM	[3]
Arg1697Trp	LMM	DCM	[31]
Gln1704Term	LMM	HCM	[3]
Leu1706Pro	LMM	Myopathy	[211]
Leu1706Val	LMM	CM	[227]
Ile1707Phe	LMM	HCM	[24]
Arg1712Trp	LMM	HCM	[197]
Arg1712Gln	LMM	HCM	[13]
His1717Gln	LMM	HCM	[228]
Gln1719Arg	LMM	HCM	[24]
Leu1723Pro	LMM	Myopathy	[229]
Ile1724Met	LMM	HCM	[3]
Lys1727Term	LMM	DCM	[31]
Asp1731Val	LMM	DCM	[56]
Ala1744Ser	LMM	Sudden cardiac death	[230]
Cys1748Tyr	LMM	Sudden cardiac death	[190]
Glu1752Lys	LMM	HCM	[231]
Glu1752Val	LMM	HCM	[17]
Glu1753Lys	LMM	HCM	[197]
Lys1757Glu	LMM	HCM	[19]
Thr1760Arg	LMM	HCM	[191]
Thr1760Met	LMM	HCM	[172]
Ala1763Thr	LMM	HCM	[25]
Met1764Lys	LMM	HCM	[3]
Met1765Lys	LMM	HCM	[6]
Ala1766Thr	LMM	CM	[65]
Glu1768Lys	LMM	HCM	[23]
Leu1769Met	LMM	HCM	[55]
Gln1773Lys	LMM	HCM	[194]
Thr1775Ile	LMM	HCM	[14]
Ser1776Gly	LMM	HCM	[197]
Ala1777Thr	LMM	HCM	[10]
His1778Tyr	LMM	HCM	[3]
Leu1779Pro	LMM	Myopathy	[208]

Arg1781Cys	LMM	HCM	[25]
Arg1781His	LMM	HCM	[6]
Met1782Val	LMM	HCM	[124]
Asp1792Gly	LMM	DCM	[17]
Leu1793Pro	LMM	Myopathy	[232]
Gln1794Lys	LMM	HCM	[233]
Gln1794Glu	LMM	DCM	[90]
Leu1797Pro	LMM	DCM	[192]
GLu1799Lys	LMM	LVNC	[74]
Glu1801Gly	LMM	DCM	[17]
Glu1801Lys	LMM	Myopathy/DCM	[61, 220]
Ala1804Thr	LMM	DCM	[234]
Gly1808Ala	LMM	DCM	[57]
Gly1808Ser	LMM	HCM	[114]
Arg1818Trp	LMM	HCM	[25]
Arg1820Gln	LMM	CM, distal myopathy	[235]
Arg1820Trp	LMM	Myopathy	[236]
Asn1824Cys	LMM	HCM	[3]
Asn1824Ser	LMM	HCM	[17]
Glu1829Gly	LMM	HCM	[20]
Arg1832Cys	LMM	DCM	[137]
Ala1834Thr	LMM	HCM	[24]
Glu1835Term	LMM	HCM	[19]
Ser1836Leu	LMM	HCM	[137]
Lys1838Glu	LMM	DCM	[84]
Ser1843Gly	LMM	HCM	[24]
Glu1844Lys	LMM	DCM	[3]
Arg1845Trp	LMM	Myopathy	[237]
Arg1846Cys	LMM	HCM	[50]
Lys1848Thr	LMM	HCM	[13]
Tyr1852Term	LMM	DCM	[84]
Thr1854Met	LMM	HCM	[23]
Thr1854Thr	LMM	DCM	[238]
Glu1856Lys	LMM	LVNC	[220]
Arg1858Met	LMM	HCM	[47]
Arg1863Trp	LMM	HCM	[3]
Arg1863Gln	LMM	DCM	[57]
Asp1869Gly	LMM	HCM	[24]
Val1875Phe	LMM	DCM	[196]
Lys1879Glu	LMM	DCM	[169]
Arg1880His	LMM	HCM	[157]
Glu1883Lys	LMM	Myopathy, HCM	[239]
Glu1886Lys	LMM	Myopathy	[220]
Glu1887Gly	LMM	Sudden cardiac death	[240]
Arg1897His	LMM	HCM	[6]
Val1899Ala	LMM	HCM	[24]
His1901Gln	LMM	DCM	[57]
His1901Leu	LMM	Myopathy	[241]
Glu1902Gln	LMM	HCM	[33]
Glu1902Lys	LMM	DCM	[56]
Ala1906Gly	LMM	DCM	[84]

Arg1909Pro	LMM	DCM	[212]
Arg1909Trp	LMM	HCM	[3]
Glu1914Lys	LMM	DCM	[90]
Val1917Phe	LMM	HCM	[3]
Asn1918Lys	LMM	Ebstein anomaly	[73]
Arg1925Cys	LMM	HCM	[3]
Arg1925Gly	LMM	LVNC	[140]
Ile1927Phe	LMM	HCM	[50]
Thr1929Met	LMM	HCM	[23]
Gly1931Cys	LMM	HCM	[99]
Thr1929Met	LMM	HCM	[23]
Term1936Leu		Myopathy	[242]
Term1936Trp		Myopathy	[243]
Term1936Tyr		Myopathy	[133]

1. Garcia-Castro, M., et al., *[Mutations in sarcomeric genes MYH7, MYBPC3, TNNT2, TNNI3, and TPM1 in patients with hypertrophic cardiomyopathy].* Rev Esp Cardiol, 2009. **62**(1): p. 48-56.
2. Perkins, B.A., et al., *Precision medicine screening using whole-genome sequencing and advanced imaging to identify disease risk in adults.* Proc Natl Acad Sci U S A, 2018. **115**(14): p. 3686-3691.
3. Walsh, R., et al., *Reassessment of Mendelian gene pathogenicity using 7,855 cardiomyopathy cases and 60,706 reference samples.* Genet Med, 2017. **19**(2): p. 192-203.
4. Seleznev, D.M., et al., *[The role of mutation in cardiac beta-myosin heavy chain gene in population of patients].* Kardiologiia, 2005. **45**(4): p. 15-20.
5. Alfares, A.A., et al., *Results of clinical genetic testing of 2,912 probands with hypertrophic cardiomyopathy: expanded panels offer limited additional sensitivity.* Genet Med, 2015. **17**(11): p. 880-8.
6. Lopes, L.R., et al., *Novel genotype-phenotype associations demonstrated by high-throughput sequencing in patients with hypertrophic cardiomyopathy.* Heart, 2015. **101**(4): p. 294-301.
7. Wang, C., et al., *A Wide and Specific Spectrum of Genetic Variants and Genotype-Phenotype Correlations Revealed by Next-Generation Sequencing in Patients with Left Ventricular Noncompaction.* J Am Heart Assoc, 2017. **6**(9).
8. Arai, S., et al., *Missense mutation of the beta-cardiac myosin heavy-chain gene in hypertrophic cardiomyopathy.* Am J Med Genet, 1995. **58**(3): p. 267-76.
9. Golbus, J.R., et al., *Population-based variation in cardiomyopathy genes.* Circ Cardiovasc Genet, 2012. **5**(4): p. 391-9.
10. Richard, P., et al., *Hypertrophic cardiomyopathy: distribution of disease genes, spectrum of mutations, and implications for a molecular diagnosis strategy.* Circulation, 2003. **107**(17): p. 2227-32.
11. Mook, O.R., et al., *Targeted sequence capture and GS-FLX Titanium sequencing of 23 hypertrophic and dilated cardiomyopathy genes: implementation into diagnostics.* J Med Genet, 2013. **50**(9): p. 614-26.
12. Kolokotronis, K., et al., *Biallelic mutation in MYH7 and MYBPC3 leads to severe cardiomyopathy with left ventricular noncompaction phenotype.* Hum Mutat, 2019. **40**(8): p. 1101-1114.
13. Zou, Y., et al., *Multiple gene mutations, not the type of mutation, are the modifier of left ventricle hypertrophy in patients with hypertrophic cardiomyopathy.* Mol Biol Rep, 2013. **40**(6): p. 3969-76.
14. Bos, J.M., et al., *Characterization of a phenotype-based genetic test prediction score for unrelated patients with hypertrophic cardiomyopathy.* Mayo Clin Proc, 2014. **89**(6): p. 727-37.
15. Nishi, H., et al., *A myosin missense mutation, not a null allele, causes familial hypertrophic cardiomyopathy.* Circulation, 1995. **91**(12): p. 2911-5.
16. Coppini, R., et al., *Clinical phenotype and outcome of hypertrophic cardiomyopathy associated with thin-filament gene mutations.* J Am Coll Cardiol, 2014. **64**(24): p. 2589-2600.

17. Waldmuller, S., et al., *Novel correlations between the genotype and the phenotype of hypertrophic and dilated cardiomyopathy: results from the German Competence Network Heart Failure.* Eur J Heart Fail, 2011. **13**(11): p. 1185-92.
18. van Waning, J.I., et al., *Genetics, Clinical Features, and Long-Term Outcome of Noncompaction Cardiomyopathy.* J Am Coll Cardiol, 2018. **71**(7): p. 711-722.
19. Wang, J., et al., *Malignant effects of multiple rare variants in sarcomere genes on the prognosis of patients with hypertrophic cardiomyopathy.* Eur J Heart Fail, 2014. **16**(9): p. 950-7.
20. Coto, E., et al., *Resequencing the whole MYH7 gene (including the intronic, promoter, and 3' UTR sequences) in hypertrophic cardiomyopathy.* J Mol Diagn, 2012. **14**(5): p. 518-24.
21. Miszalski-Jamka, K., et al., *Novel Genetic Triggers and Genotype-Phenotype Correlations in Patients With Left Ventricular Noncompaction.* Circ Cardiovasc Genet, 2017. **10**(4).
22. Alejandra Restrepo-Cordoba, M., et al., *Usefulness of Genetic Testing in Hypertrophic Cardiomyopathy: an Analysis Using Real-World Data.* J Cardiovasc Transl Res, 2017. **10**(1): p. 35-46.
23. Van Driest, S.L., et al., *Comprehensive analysis of the beta-myosin heavy chain gene in 389 unrelated patients with hypertrophic cardiomyopathy.* J Am Coll Cardiol, 2004. **44**(3): p. 602-10.
24. Homburger, J.R., et al., *Multidimensional structure-function relationships in human beta-cardiac myosin from population-scale genetic variation.* Proc Natl Acad Sci U S A, 2016. **113**(24): p. 6701-6.
25. Berge, K.E. and T.P. Leren, *Genetics of hypertrophic cardiomyopathy in Norway.* Clin Genet, 2013.
26. Rayment, I., et al., *Structural interpretation of the mutations in the beta-cardiac myosin that have been implicated in familial hypertrophic cardiomyopathy.* Proc Natl Acad Sci U S A, 1995. **92**(9): p. 3864-8.
27. Richard, P., et al., *Targeted panel sequencing in adult patients with left ventricular non-compaction reveals a large genetic heterogeneity.* Clin Genet, 2019. **95**(3): p. 356-367.
28. Mohiddin, S.A., et al., *Utility of genetic screening in hypertrophic cardiomyopathy: prevalence and significance of novel and double (homozygous and heterozygous) beta-myosin mutations.* Genet Test, 2003. **7**(1): p. 21-7.
29. Erdmann, J., et al., *Mutation spectrum in a large cohort of unrelated consecutive patients with hypertrophic cardiomyopathy.* Clin Genet, 2003. **64**(4): p. 339-49.
30. Kimura, A., et al., *Molecular etiology of idiopathic cardiomyopathy in Asian populations.* J Cardiol, 2001. **37 Suppl 1**: p. 139-46.
31. Haas, J., et al., *Atlas of the clinical genetics of human dilated cardiomyopathy.* Eur Heart J, 2015. **36**(18): p. 1123-35a.
32. Ingles, J., et al., *Compound and double mutations in patients with hypertrophic cardiomyopathy: implications for genetic testing and counselling.* J Med Genet, 2005. **42**(10): p. e59.
33. Chiou, K.R., C.T. Chu, and M.J. Charng, *Detection of mutations in symptomatic patients with hypertrophic cardiomyopathy in Taiwan.* J Cardiol, 2015. **65**(3): p. 250-6.
34. Punetha, J., et al., *Targeted Re-Sequencing Emulsion PCR Panel for Myopathies: Results in 94 Cases.* J Neuromuscul Dis, 2016. **3**(2): p. 209-225.
35. Meder, B., et al., *Targeted next-generation sequencing for the molecular genetic diagnostics of cardiomyopathies.* Circ Cardiovasc Genet, 2011. **4**(2): p. 110-22.
36. Bainbridge, M.N., et al., *Loss of Function Mutations in NNT Are Associated With Left Ventricular Noncompaction.* Circ Cardiovasc Genet, 2015. **8**(4): p. 544-52.
37. Pan, S., et al., *Cardiac structural and sarcomere genes associated with cardiomyopathy exhibit marked intolerance of genetic variation.* Circ Cardiovasc Genet, 2012. **5**(6): p. 602-10.
38. Kassem, H., et al., *Early results of sarcomeric gene screening from the Egyptian National BA-HCM Program.* J Cardiovasc Transl Res, 2013. **6**(1): p. 65-80.
39. Millat, G., et al., *Development of a high resolution melting method for the detection of genetic variations in hypertrophic cardiomyopathy.* Clin Chim Acta, 2010. **411**(23-24): p. 1983-91.
40. Mademont-Soler, I., et al., *Additional value of screening for minor genes and copy number variants in hypertrophic cardiomyopathy.* PLoS One, 2017. **12**(8): p. e0181465.
41. Miller, E.M., et al., *Genetic Testing in Pediatric Left Ventricular Noncompaction.* Circ Cardiovasc Genet, 2017. **10**(6).
42. Bundgaard, H., et al., *Familial hypertrophic cardiomyopathy associated with a novel missense mutation affecting the ATP-binding region of the cardiac beta-myosin heavy chain.* J Mol Cell Cardiol, 1999. **31**(4): p. 745-50.

43. Iascone, M.R., D. Marchetti, and P. Ferrazzi, *Gene symbol: MYH7.* Hum Genet, 2007. **120**(6): p. 916.
44. Woo, A., et al., *Mutations of the beta myosin heavy chain gene in hypertrophic cardiomyopathy: critical functional sites determine prognosis.* Heart, 2003. **89**(10): p. 1179-85.
45. Fujino, N., et al., *Impact of systolic dysfunction in genotyped hypertrophic cardiomyopathy.* Clin Cardiol, 2013. **36**(3): p. 160-5.
46. Villard, E., et al., *Mutation screening in dilated cardiomyopathy: prominent role of the beta myosin heavy chain gene.* Eur Heart J, 2005. **26**(8): p. 794-803.
47. Santos, S., et al., *High resolution melting: improvements in the genetic diagnosis of hypertrophic cardiomyopathy in a Portuguese cohort.* BMC Med Genet, 2012. **13**: p. 17.
48. Hazebroek, M.R., et al., *Prevalence of Pathogenic Gene Mutations and Prognosis Do Not Differ in Isolated Left Ventricular Dysfunction Compared With Dilated Cardiomyopathy.* Circ Heart Fail, 2018. **11**(3): p. e004682.
49. Waldmuller, S., et al., *Array-based resequencing assay for mutations causing hypertrophic cardiomyopathy.* Clin Chem, 2008. **54**(4): p. 682-7.
50. Millat, G., et al., *Prevalence and spectrum of mutations in a cohort of 192 unrelated patients with hypertrophic cardiomyopathy.* Eur J Med Genet, 2010. **53**(5): p. 261-7.
51. Daehmlow, S., et al., *Novel mutations in sarcomeric protein genes in dilated cardiomyopathy.* Biochem Biophys Res Commun, 2002. **298**(1): p. 116-20.
52. Bottillo, I., et al., *Molecular analysis of sarcomeric and non-sarcomeric genes in patients with hypertrophic cardiomyopathy.* Gene, 2016. **577**(2): p. 227-35.
53. Yu, B., et al., *Denaturing high performance liquid chromatography: high throughput mutation screening in familial hypertrophic cardiomyopathy and SNP genotyping in motor neurone disease.* J Clin Pathol, 2005. **58**(5): p. 479-85.
54. Dufour, C., et al., *Identification of a mutation near a functional site of the beta cardiac myosin heavy chain gene in a family with hypertrophic cardiomyopathy.* J Mol Cell Cardiol, 1994. **26**(9): p. 1241-7.
55. Girolami, F., et al., *A molecular screening strategy based on beta-myosin heavy chain, cardiac myosin binding protein C and troponin T genes in Italian patients with hypertrophic cardiomyopathy.* J Cardiovasc Med (Hagerstown), 2006. **7**(8): p. 601-7.
56. Zimmerman, R.S., et al., *A novel custom resequencing array for dilated cardiomyopathy.* Genet Med, 2010. **12**(5): p. 268-78.
57. Hershberger, R.E., et al., *Coding sequence mutations identified in MYH7, TNNT2, SCN5A, CSRP3, LBD3, and TCAP from 313 patients with familial or idiopathic dilated cardiomyopathy.* Clin Transl Sci, 2008. **1**(1): p. 21-6.
58. Hauser, N.S., et al., *Experience with genomic sequencing in pediatric patients with congenital cardiac defects in a large community hospital.* Mol Genet Genomic Med, 2018. **6**(2): p. 200-212.
59. Kubo, T., et al., *Genetic screening and double mutation in Japanese patients with hypertrophic cardiomyopathy.* Circ J, 2011. **75**(11): p. 2654-9.
60. Arad, M., et al., *Gene mutations in apical hypertrophic cardiomyopathy.* Circulation, 2005. **112**(18): p. 2805-11.
61. Pugh, T.J., et al., *The landscape of genetic variation in dilated cardiomyopathy as surveyed by clinical DNA sequencing.* Genet Med, 2014. **16**(8): p. 601-8.
62. Rosenzweig, A., et al., *Preclinical diagnosis of familial hypertrophic cardiomyopathy by genetic analysis of blood lymphocytes.* N Engl J Med, 1991. **325**(25): p. 1753-60.
63. Tian, T., et al., *A low prevalence of sarcomeric gene variants in a Chinese cohort with left ventricular non-compaction.* Heart Vessels, 2015. **30**(2): p. 258-64.
64. Kaski, J.P., et al., *Prevalence of sarcomere protein gene mutations in preadolescent children with hypertrophic cardiomyopathy.* Circ Cardiovasc Genet, 2009. **2**(5): p. 436-41.
65. Klaassen, S., et al., *Mutations in sarcomere protein genes in left ventricular noncompaction.* Circulation, 2008. **117**(22): p. 2893-901.
66. Yu, B.L., et al., *A novel MYH7 mutation in a family with cardiomyopathy presenting with restrictive physiology and varying degrees of left ventricle hypertrophy.* Eur Heart J, 2015. **36**(3): p. 178.
67. Fananapazir, L., et al., *Missense mutations in the beta-myosin heavy-chain gene cause central core disease in hypertrophic cardiomyopathy.* Proc Natl Acad Sci U S A, 1993. **90**(9): p. 3993-7.

68. Tesson, F., et al., *Genotype-phenotype analysis in four families with mutations in beta-myosin heavy chain gene responsible for familial hypertrophic cardiomyopathy.* Hum Mutat, 1998. **12**(6): p. 385-92.
69. Rai, T.S., et al., *Genotype phenotype correlations of cardiac beta-myosin heavy chain mutations in Indian patients with hypertrophic and dilated cardiomyopathy.* Mol Cell Biochem, 2009. **321**(1-2): p. 189-96.
70. Olivotto, I., et al., *Myofilament protein gene mutation screening and outcome of patients with hypertrophic cardiomyopathy.* Mayo Clin Proc, 2008. **83**(6): p. 630-8.
71. Daoud, H., et al., *Genetic Diagnostic Testing for Inherited Cardiomyopathies: Considerations for Offering Multi-Gene Tests in a Health Care Setting.* J Mol Diagn, 2019. **21**(3): p. 437-448.
72. Budde, B.S., et al., *Noncompaction of the ventricular myocardium is associated with a de novo mutation in the beta-myosin heavy chain gene.* PLoS One, 2007. **2**(12): p. e1362.
73. Postma, A.V., et al., *Mutations in the sarcomere gene MYH7 in Ebstein anomaly.* Circ Cardiovasc Genet, 2011. **4**(1): p. 43-50.
74. Takasaki, A., et al., *Sarcomere gene variants act as a genetic trigger underlying the development of left ventricular noncompaction.* Pediatr Res, 2018. **84**(5): p. 733-742.
75. Lakdawala, N.K., et al., *Electrocardiographic features of sarcomere mutation carriers with and without clinically overt hypertrophic cardiomyopathy.* Am J Cardiol, 2011. **108**(11): p. 1606-13.
76. Hoedemaekers, Y.M., et al., *Cardiac beta-myosin heavy chain defects in two families with non-compaction cardiomyopathy: linking non-compaction to hypertrophic, restrictive, and dilated cardiomyopathies.* Eur Heart J, 2007. **28**(22): p. 2732-7.
77. Burns, C., et al., *Multiple Gene Variants in Hypertrophic Cardiomyopathy in the Era of Next-Generation Sequencing.* Circ Cardiovasc Genet, 2017. **10**(4).
78. Havndrup, O., et al., *Outcome of clinical versus genetic family screening in hypertrophic cardiomyopathy with focus on cardiac beta-myosin gene mutations.* Cardiovasc Res, 2003. **57**(2): p. 347-57.
79. Bashyam, M.D., et al., *A low prevalence of MYH7/MYBPC3 mutations among familial hypertrophic cardiomyopathy patients in India.* Mol Cell Biochem, 2012. **360**(1-2): p. 373-82.
80. Helms, A.S., et al., *Sarcomere mutation-specific expression patterns in human hypertrophic cardiomyopathy.* Circ Cardiovasc Genet, 2014. **7**(4): p. 434-43.
81. Andreasen, C., et al., *New population-based exome data are questioning the pathogenicity of previously cardiomyopathy-associated genetic variants.* Eur J Hum Genet, 2013. **21**(9): p. 918-28.
82. Norrish, G., et al., *Yield of Clinical Screening for Hypertrophic Cardiomyopathy in Child First-Degree Relatives.* Circulation, 2019. **140**(3): p. 184-192.
83. Valente, A.M., et al., *Comparison of echocardiographic and cardiac magnetic resonance imaging in hypertrophic cardiomyopathy sarcomere mutation carriers without left ventricular hypertrophy.* Circ Cardiovasc Genet, 2013. **6**(3): p. 230-7.
84. Horvat, C., et al., *A gene-centric strategy for identifying disease-causing rare variants in dilated cardiomyopathy.* Genet Med, 2019. **21**(1): p. 133-143.
85. Jeschke, B., et al., *A high risk phenotype of hypertrophic cardiomyopathy associated with a compound genotype of two mutated beta-myosin heavy chain genes.* Hum Genet, 1998. **102**(3): p. 299-304.
86. Hirono, K., et al., *Familial Ebstein's anomaly, left ventricular noncompaction, and ventricular septal defect associated with an MYH7 mutation.* J Thorac Cardiovasc Surg, 2014. **148**(5): p. e223-6.
87. Dellefave, L.M., et al., *Sarcomere mutations in cardiomyopathy with left ventricular hypertrabeculation.* Circ Cardiovasc Genet, 2009. **2**(5): p. 442-9.
88. Boda, U., et al., *Novel mutations in beta-myosin heavy chain, actin and troponin-I genes associated with dilated cardiomyopathy in Indian population.* J Genet, 2009. **88**(3): p. 373-7.
89. Kuang, S.Q., et al., *Identification of a novel missense mutation in the cardiac beta-myosin heavy chain gene in a Chinese patient with sporadic hypertrophic cardiomyopathy.* J Mol Cell Cardiol, 1996. **28**(9): p. 1879-83.
90. Lakdawala, N.K., et al., *Genetic testing for dilated cardiomyopathy in clinical practice.* J Card Fail, 2012. **18**(4): p. 296-303.
91. Gifford, C.A., et al., *Oligogenic inheritance of a human heart disease involving a genetic modifier.* Science, 2019. **364**(6443): p. 865-870.
92. Laredo, R., et al., *[Beta-myosin heavy-chain gene mutations in patients with hypertrophic cardiomyopathy].* Rev Esp Cardiol, 2006. **59**(10): p. 1008-18.

93. Blanchard, E., et al., *Altered crossbridge kinetics in the alphaMHC403/+ mouse model of familial hypertrophic cardiomyopathy.* Circ Res, 1999. **84**(4): p. 475-83.
94. Dausse, E., et al., *Familial hypertrophic cardiomyopathy. Microsatellite haplotyping and identification of a hot spot for mutations in the beta-myosin heavy chain gene.* J Clin Invest, 1993. **92**(6): p. 2807-13.
95. Greber-Platzer, S., et al., *Beta-myosin heavy chain gene mutations and hypertrophic cardiomyopathy in Austrian children.* J Mol Cell Cardiol, 2001. **33**(1): p. 141-8.
96. Guo, Q., et al., *Exome sequencing identifies a novel MYH7 p.G407C mutation responsible for familial hypertrophic cardiomyopathy.* DNA Cell Biol, 2014. **33**(10): p. 699-704.
97. Lu, C., et al., *Molecular analysis of inherited cardiomyopathy using next generation semiconductor sequencing technologies.* J Transl Med, 2018. **16**(1): p. 241.
98. Song, L., et al., *Mutations profile in Chinese patients with hypertrophic cardiomyopathy.* Clin Chim Acta, 2005. **351**(1-2): p. 209-16.
99. Nunez, L., et al., *Somatic MYH7, MYBPC3, TPM1, TNNT2 and TNNI3 mutations in sporadic hypertrophic cardiomyopathy.* Circ J, 2013. **77**(9): p. 2358-65.
100. Kuhnisch, J., et al., *Targeted panel sequencing in pediatric primary cardiomyopathy supports a critical role of TNNI3.* Clin Genet, 2019. **96**(6): p. 549-559.
101. Morner, S., et al., *Identification of the genotypes causing hypertrophic cardiomyopathy in northern Sweden.* J Mol Cell Cardiol, 2003. **35**(7): p. 841-9.
102. Pagola-Lorz, I., et al., *Epidemiological study and genetic characterization of inherited muscle diseases in a northern Spanish region.* Orphanet J Rare Dis, 2019. **14**(1): p. 276.
103. Basu, R., et al., *Novel mutation in exon 14 of the sarcomere gene MYH7 in familial left ventricular noncompaction with bicuspid aortic valve.* Circ Heart Fail, 2014. **7**(6): p. 1059-62.
104. Darin, N., et al., *New skeletal myopathy and cardiomyopathy associated with a missense mutation in MYH7.* Neurology, 2007. **68**(23): p. 2041-2.
105. Kamisago, M., et al., *Sarcomere protein gene mutations and inherited heart disease: a beta-cardiac myosin heavy chain mutation causing endocardial fibroelastosis and heart failure.* Novartis Found Symp, 2006. **274**: p. 176-89; discussion 189-95, 272-6.
106. Roncarati, R., et al., *Unexpectedly low mutation rates in beta-myosin heavy chain and cardiac myosin binding protein genes in Italian patients with hypertrophic cardiomyopathy.* J Cell Physiol, 2011. **226**(11): p. 2894-900.
107. Liu, W., et al., *Mutation spectrum in a large cohort of unrelated Chinese patients with hypertrophic cardiomyopathy.* Am J Cardiol, 2013. **112**(4): p. 585-9.
108. Arbustini, E., et al., *Coexistence of mitochondrial DNA and beta myosin heavy chain mutations in hypertrophic cardiomyopathy with late congestive heart failure.* Heart, 1998. **80**(6): p. 548-58.
109. Watkins, H., et al., *Characteristics and prognostic implications of myosin missense mutations in familial hypertrophic cardiomyopathy.* N Engl J Med, 1992. **326**(17): p. 1108-14.
110. Frazier, A., et al., *Familial hypertrophic cardiomyopathy associated with cardiac beta-myosin heavy chain and troponin I mutations.* Pediatr Cardiol, 2008. **29**(4): p. 846-50.
111. Mamelona, J., et al., *A novel missense mutation in the MYH7 gene causes an uncharacteristic phenotype of myosin storage myopathy: a case report.* BMC Med Genet, 2019. **20**(1): p. 78.
112. Curila, K., et al., *Spectrum and clinical manifestations of mutations in genes responsible for hypertrophic cardiomyopathy.* Acta Cardiol, 2012. **67**(1): p. 23-9.
113. Richard, P., et al., *Double heterozygosity for mutations in the beta-myosin heavy chain and in the cardiac myosin binding protein C genes in a family with hypertrophic cardiomyopathy.* J Med Genet, 1999. **36**(7): p. 542-5.
114. Daher, J., et al., *Screening of MYH7, MYBPC3, and TNNT2 genes in Brazilian patients with hypertrophic cardiomyopathy.* Am Heart J, 2013. **166**: p. 775-782.
115. Ross, S.B., et al., *Burden of Recurrent and Ancestral Mutations in Families With Hypertrophic Cardiomyopathy.* Circ Cardiovasc Genet, 2017. **10**(3).
116. Meyer, T., et al., *Detection of a large duplication mutation in the myosin-binding protein C3 gene in a case of hypertrophic cardiomyopathy.* Gene, 2013. **527**(1): p. 416-20.
117. Yang, J., et al., *Whole-exome sequencing identify a new mutation of MYH7 in a Chinese family with left ventricular noncompaction.* Gene, 2015. **558**(1): p. 138-42.
118. Moolman-Smook, J.C., et al., *The origins of hypertrophic cardiomyopathy-causing mutations in two South African subpopulations: a unique profile of both independent and founder events.* Am J Hum Genet, 1999. **65**(5): p. 1308-20.
119. Perrot, A., et al., *Prevalence of cardiac beta-myosin heavy chain gene mutations in patients with hypertrophic cardiomyopathy.* J Mol Med (Berl), 2005. **83**(6): p. 468-77.

120. Anan, R., et al., *Prognostic implications of novel beta cardiac myosin heavy chain gene mutations that cause familial hypertrophic cardiomyopathy.* J Clin Invest, 1994. **93**(1): p. 280-5.
121. Mora, R., et al., *[Hypertrophic cardiomyopathy: infrequent mutation of the cardiac beta-myosin heavy-chain gene].* Rev Esp Cardiol, 2006. **59**(8): p. 846-9.
122. Otsuka, H., et al., *Prevalence and distribution of sarcomeric gene mutations in Japanese patients with familial hypertrophic cardiomyopathy.* Circ J, 2012. **76**(2): p. 453-61.
123. Nanni, L., et al., *Hypertrophic cardiomyopathy: two homozygous cases with "typical" hypertrophic cardiomyopathy and three new mutations in cases with progression to dilated cardiomyopathy.* Biochem Biophys Res Commun, 2003. **309**(2): p. 391-8.
124. Murphy, S.L., et al., *Evaluation of the Mayo Clinic Phenotype-Based Genotype Predictor Score in Patients with Clinically Diagnosed Hypertrophic Cardiomyopathy.* J Cardiovasc Transl Res, 2016. **9**(2): p. 153-61.
125. Kaneda, T., et al., *A novel beta-myosin heavy chain gene mutation, p.Met531Arg, identified in isolated left ventricular non-compaction in humans, results in left ventricular hypertrophy that progresses to dilation in a mouse model.* Clin Sci (Lond), 2008. **114**(6): p. 431-40.
126. Hayashi, T., et al., *Genetic background of Japanese patients with pediatric hypertrophic and restrictive cardiomyopathy.* J Hum Genet, 2018. **63**(9): p. 989-996.
127. Agarwal, A., et al., *Clinical application of WHF-MOGE(S) classification for hypertrophic cardiomyopathy.* Glob Heart, 2015. **10**(3): p. 209-19.
128. Cuenca, S., et al., *Genetic basis of familial dilated cardiomyopathy patients undergoing heart transplantation.* J Heart Lung Transplant, 2016. **35**(5): p. 625-35.
129. Nomura, Y., et al., *A novel MYH7 gene mutation in a fetus with left ventricular noncompaction.* Can J Cardiol, 2015. **31**(1): p. 103.e1-3.
130. Yang, Q., et al., *[Analysis of genotype-phenotype correlation for a novel MYH7-D554Y mutation identified in an ethnic Han Chinese pedigree affected with hypertrophic cardiomyopathy].* Zhonghua Yi Xue Yi Chuan Xue Za Zhi, 2018. **35**(5): p. 667-671.
131. Captur, G., et al., *Prediction of sarcomere mutations in subclinical hypertrophic cardiomyopathy.* Circ Cardiovasc Imaging, 2014. **7**(6): p. 863-71.
132. Marian, A.J. and R. Roberts, *Recent advances in the molecular genetics of hypertrophic cardiomyopathy.* Circulation, 1995. **92**(5): p. 1336-47.
133. Fiorillo, C., et al., *MYH7-related myopathies: clinical, histopathological and imaging findings in a cohort of Italian patients.* Orphanet J Rare Dis, 2016. **11**(1): p. 91.
134. Nishi, H., et al., *Novel missense mutation in cardiac beta myosin heavy chain gene found in a Japanese patient with hypertrophic cardiomyopathy.* Biochem Biophys Res Commun, 1992. **188**(1): p. 379-87.
135. Gomez, J., et al., *Mutation analysis of the main hypertrophic cardiomyopathy genes using multiplex amplification and semiconductor next-generation sequencing.* Circ J, 2014. **78**(12): p. 2963-71.
136. Ohsuzu, F., et al., *Hypertrophic obstructive cardiomyopathy due to a novel T-to-A transition at codon 624 in the beta-myosin heavy chain (beta-MHC) gene possibly related to the sudden death.* Int J Cardiol, 1997. **62**(3): p. 203-9.
137. Moller, D.V., et al., *The role of sarcomere gene mutations in patients with idiopathic dilated cardiomyopathy.* Eur J Hum Genet, 2009. **17**(10): p. 1241-9.
138. Ho, C.Y., et al., *Assessment of diastolic function with Doppler tissue imaging to predict genotype in preclinical hypertrophic cardiomyopathy.* Circulation, 2002. **105**(25): p. 2992-7.
139. Hill, M.G., et al., *Intrauterine Treatment of a Fetus with Familial Hypertrophic Cardiomyopathy Secondary to MYH7 Mutation.* Pediatr Cardiol, 2015. **36**(8): p. 1774-7.
140. Hoedemaekers, Y.M., et al., *The importance of genetic counseling, DNA diagnostics, and cardiologic family screening in left ventricular noncompaction cardiomyopathy.* Circ Cardiovasc Genet, 2010. **3**(3): p. 232-9.
141. van Spaendonck-Zwarts, K.Y., et al., *Genetic analysis in 418 index patients with idiopathic dilated cardiomyopathy: overview of 10 years' experience.* Eur J Heart Fail, 2013. **15**(6): p. 628-36.
142. Gruver, E.J., et al., *Familial hypertrophic cardiomyopathy and atrial fibrillation caused by Arg663His beta-cardiac myosin heavy chain mutation.* Am J Cardiol, 1999. **83**(12A): p. 13H-18H.
143. Rubegni, A., et al., *Next-generation sequencing approach to hyperCKemia: A 2-year cohort study.* Neurol Genet, 2019. **5**(5): p. e352.

144. Andersen, P.S., et al., *Adult-onset familial hypertrophic cardiomyopathy caused by a novel mutation, R694C, in the MYH7 gene.* Clin Genet, 1999. **56**(3): p. 244-6.
145. Zheng, D.D., et al., *Mutations in the beta-myosin heavy chain gene in southern Chinese families with hypertrophic cardiomyopathy.* J Int Med Res, 2010. **38**(3): p. 810-20.
146. Jaaskelainen, P., et al., *The cardiac beta-myosin heavy chain gene is not the predominant gene for hypertrophic cardiomyopathy in the Finnish population.* J Am Coll Cardiol, 1998. **32**(6): p. 1709-16.
147. Cecconi, M., et al., *Targeted next-generation sequencing helps to decipher the genetic and phenotypic heterogeneity of hypertrophic cardiomyopathy.* Int J Mol Med, 2016. **38**(4): p. 1111-24.
148. Ochoa, J.P., et al., *Formin Homology 2 Domain Containing 3 (FHOD3) Is a Genetic Basis for Hypertrophic Cardiomyopathy.* J Am Coll Cardiol, 2018. **72**(20): p. 2457-2467.
149. Kindel, S.J., et al., *Pediatric cardiomyopathy: importance of genetic and metabolic evaluation.* J Card Fail, 2012. **18**(5): p. 396-403.
150. Sakthivel, T., I. Toth, and A.T. Florence, *Distribution of a lipidic 2.5 nm diameter dendrimer carrier after oral administration.* Int J Pharm, 1999. **183**(1): p. 51-5.
151. Arad, M., et al., *Merits and pitfalls of genetic testing in a hypertrophic cardiomyopathy clinic.* Isr Med Assoc J, 2014. **16**(11): p. 707-13.
152. Consevage, M.W., et al., *A new missense mutation, Arg719Gln, in the beta-cardiac heavy chain myosin gene of patients with familial hypertrophic cardiomyopathy.* Hum Mol Genet, 1994. **3**(6): p. 1025-6.
153. Enjuto, M., et al., *Malignant hypertrophic cardiomyopathy caused by the Arg723Gly mutation in beta-myosin heavy chain gene.* J Mol Cell Cardiol, 2000. **32**(12): p. 2307-13.
154. Blair, E., et al., *Mutations in cis can confound genotype-phenotype correlations in hypertrophic cardiomyopathy.* J Med Genet, 2001. **38**(6): p. 385-8.
155. Garcia-Giustiniani, D., et al., *Phenotype and prognostic correlations of the converter region mutations affecting the beta myosin heavy chain.* Heart, 2015. **101**(13): p. 1047-53.
156. Kato, M., et al., *Altered actin binding with myosin mutation in hypertrophic cardiomyopathy and sudden death.* Lancet, 1995. **345**(8959): p. 1247.
157. Jaafar, N., et al., *Spectrum of Mutations in Hypertrophic Cardiomyopathy Genes Among Tunisian Patients.* Genet Test Mol Biomarkers, 2016. **20**(11): p. 674-679.
158. Homayoun, H., et al., *Novel mutation in MYH7 gene associated with distal myopathy and cardiomyopathy.* Neuromuscul Disord, 2011. **21**(3): p. 219-22.
159. Davis, J.S., et al., *The overall pattern of cardiac contraction depends on a spatial gradient of myosin regulatory light chain phosphorylation.* Cell, 2001. **107**(5): p. 631-41.
160. Lionel, A.C., et al., *Improved diagnostic yield compared with targeted gene sequencing panels suggests a role for whole-genome sequencing as a first-tier genetic test.* Genet Med, 2018. **20**(4): p. 435-443.
161. Ntusi, N.A., et al., *Clinical features, spectrum of causal genetic mutations and outcome of hypertrophic cardiomyopathy in South Africans.* Cardiovasc J Afr, 2016. **27**(3): p. 152-158.
162. Morita, H., et al., *Shared genetic causes of cardiac hypertrophy in children and adults.* N Engl J Med, 2008. **358**(18): p. 1899-908.
163. Moric, E., et al., *Three novel mutations in exon 21 encoding beta-cardiac myosin heavy chain.* J Appl Genet, 2003. **44**(1): p. 103-9.
164. Harada, H., et al., *A missense mutation of cardiac beta-myosin heavy chain gene linked to familial hypertrophic cardiomyopathy in affected Japanese families.* Biochem Biophys Res Commun, 1993. **194**(2): p. 791-8.
165. Moolman, J.C., P.A. Brink, and V.A. Corfield, *Identification of a novel Ala797Thr mutation in exon 21 of the beta-myosin heavy chain gene in hypertrophic cardiomyopathy.* Hum Mutat, 1995. **6**(2): p. 197-8.
166. Okada, S., et al., *A novel de novo mutation of beta-cardiac myosin heavy chain gene found in a twelve-year-old boy with hypertrophic cardiomyopathy.* J Genet, 2014. **93**(2): p. 557-60.
167. Karam, S., et al., *A de novo mutation of the beta cardiac myosin heavy chain gene in an infantile restrictive cardiomyopathy.* Congenit Heart Dis, 2008. **3**(2): p. 138-43.
168. Kostareva, A., et al., *Genetic Spectrum of Idiopathic Restrictive Cardiomyopathy Uncovered by Next-Generation Sequencing.* PLoS One, 2016. **11**(9): p. e0163362.
169. Vasilescu, C., et al., *Genetic Basis of Severe Childhood-Onset Cardiomyopathies.* J Am Coll Cardiol, 2018. **72**(19): p. 2324-2338.

170. Maron, B.J., M.S. Maron, and C. Semsarian, *Double or compound sarcomere mutations in hypertrophic cardiomyopathy: a potential link to sudden death in the absence of conventional risk factors.* Heart Rhythm, 2012. **9**(1): p. 57-63.
171. Garcia-Pavia, P., et al., *Genetic basis of end-stage hypertrophic cardiomyopathy.* Eur J Heart Fail, 2011. **13**(11): p. 1193-201.
172. Fokstuen, S., et al., *A DNA resequencing array for pathogenic mutation detection in hypertrophic cardiomyopathy.* Hum Mutat, 2008. **29**(6): p. 879-85.
173. Shanks, G.W., et al., *Genomic Triangulation and Coverage Analysis in Whole-Exome Sequencing-Based Molecular Autopsies.* Circ Cardiovasc Genet, 2017. **10**(5).
174. Richard, P., et al., *Homozygotes for a R869G mutation in the beta -myosin heavy chain gene have a severe form of familial hypertrophic cardiomyopathy.* J Mol Cell Cardiol, 2000. **32**(8): p. 1575-83.
175. Anan, R., H. Shono, and C. Tei, *Novel cardiac beta-myosin heavy chain gene missense mutations (R869C and R870C) that cause familial hypertrophic cardiomyopathy.* Hum Mutat, 2000. **15**(6): p. 584.
176. Watkins, H., J.G. Seidman, and C.E. Seidman, *Familial hypertrophic cardiomyopathy: a genetic model of cardiac hypertrophy.* Hum Mol Genet, 1995. **4 Spec No**: p. 1721-7.
177. Cuda, G., et al., *The in vitro motility activity of beta-cardiac myosin depends on the nature of the beta-myosin heavy chain gene mutation in hypertrophic cardiomyopathy.* J Muscle Res Cell Motil, 1997. **18**(3): p. 275-83.
178. Minoche, A.E., et al., *Genome sequencing as a first-line genetic test in familial dilated cardiomyopathy.* Genet Med, 2019. **21**(3): p. 650-662.
179. Ho, C.Y., et al., *Echocardiographic strain imaging to assess early and late consequences of sarcomere mutations in hypertrophic cardiomyopathy.* Circ Cardiovasc Genet, 2009. **2**(4): p. 314-21.
180. Zhao, Y., et al., *Identification of novel mutations including a double mutation in patients with inherited cardiomyopathy by a targeted sequencing approach using the Ion Torrent PGM system.* Int J Mol Med, 2016. **37**(6): p. 1511-20.
181. van der Zwaag, P.A., et al., *Haplotype sharing test maps genes for familial cardiomyopathies.* Clin Genet, 2011. **79**(5): p. 459-67.
182. Hoedemaekers, Y.M., et al., *Prenatal ultrasound diagnosis of MYH7 non-compaction cardiomyopathy.* Ultrasound Obstet Gynecol, 2013. **41**(3): p. 336-9.
183. Zigova, M., et al., *Finding the candidate sequence variants for diagnosis of hypertrophic cardiomyopathy in East Slovak patients.* J Clin Lab Anal, 2018. **32**(3).
184. Akinrinade, O., et al., *Genetics and genotype-phenotype correlations in Finnish patients with dilated cardiomyopathy.* Eur Heart J, 2015. **36**(34): p. 2327-37.
185. Michels, M., et al., *Disease penetrance and risk stratification for sudden cardiac death in asymptomatic hypertrophic cardiomyopathy mutation carriers.* Eur Heart J, 2009. **30**(21): p. 2593-8.
186. Rubattu, S., et al., *A Next-Generation Sequencing Approach to Identify Gene Mutations in Early- and Late-Onset Hypertrophic Cardiomyopathy Patients of an Italian Cohort.* Int J Mol Sci, 2016. **17**(8).
187. Karkkainen, S., et al., *Two novel mutations in the beta-myosin heavy chain gene associated with dilated cardiomyopathy.* Eur J Heart Fail, 2004. **6**(7): p. 861-8.
188. Girolami, F., et al., *Clinical features and outcome of hypertrophic cardiomyopathy associated with triple sarcomere protein gene mutations.* J Am Coll Cardiol, 2010. **55**(14): p. 1444-53.
189. Suktitipat, B., et al., *Molecular investigation by whole exome sequencing revealed a high proportion of pathogenic variants among Thai victims of sudden unexpected death syndrome.* PLoS One, 2017. **12**(7): p. e0180056.
190. Stepien-Wojno, M., et al., *Sudden cardiac arrest in patients without overt heart disease: a limited value of next generation sequencing.* Pol Arch Intern Med, 2018. **128**(12): p. 721-730.
191. Teramoto, R., et al., *Late Gadolinium Enhancement for Prediction of Mutation-Positive Hypertrophic Cardiomyopathy on the Basis of Panel-Wide Sequencing.* Circ J, 2018. **82**(4): p. 1139-1148.
192. Klauke, B., et al., *High proportion of genetic cases in patients with advanced cardiomyopathy including a novel homozygous Plakophilin 2-gene mutation.* PLoS One, 2017. **12**(12): p. e0189489.
193. Cann, F., et al., *Phenotype-driven molecular autopsy for sudden cardiac death.* Clin Genet, 2017. **91**(1): p. 22-29.

194. Viswanathan, S.K., et al., *Hypertrophic cardiomyopathy clinical phenotype is independent of gene mutation and mutation dosage.* PLoS One, 2017. **12**(11): p. e0187948.
195. Leung, G.K.C., et al., *Identifying the genetic causes for prenatally diagnosed structural congenital anomalies (SCAs) by whole-exome sequencing (WES).* BMC Med Genomics, 2018. **11**(1): p. 93.
196. Sousa, A., et al., *Molecular characterization of Portuguese patients with dilated cardiomyopathy.* Rev Port Cardiol, 2019. **38**(2): p. 129-139.
197. Hougs, L., et al., *One third of Danish hypertrophic cardiomyopathy patients with MYH7 mutations have mutations [corrected] in MYH7 rod region.* Eur J Hum Genet, 2005. **13**(2): p. 161-5.
198. Marsiglia, J.D., et al., *Screening of MYH7, MYBPC3, and TNNT2 genes in Brazilian patients with hypertrophic cardiomyopathy.* Am Heart J, 2013. **166**(4): p. 775-82.
199. Miller, E.M., Y. Wang, and S.M. Ware, *Uptake of cardiac screening and genetic testing among hypertrophic and dilated cardiomyopathy families.* J Genet Couns, 2013. **22**(2): p. 258-67.
200. Blair, E., et al., *Mutations of the light meromyosin domain of the beta-myosin heavy chain rod in hypertrophic cardiomyopathy.* Circ Res, 2002. **90**(3): p. 263-9.
201. Bortot, B., et al., *High-throughput genotyping robot-assisted method for mutation detection in patients with hypertrophic cardiomyopathy.* Diagn Mol Pathol, 2011. **20**(3): p. 175-9.
202. Feinstein-Linial, M., et al., *Two novel MYH7 proline substitutions cause Laing Distal Myopathy-like phenotypes with variable expressivity and neck extensor contracture.* BMC Med Genet, 2016. **17**(1): p. 57.
203. Astrea, G., et al., *Myoimaging in the NGS era: the discovery of a novel mutation in MYH7 in a family with distal myopathy and core-like features--a case report.* BMC Med Genet, 2016. **17**: p. 25.
204. Park, J.M., et al., *A novel MYH7 mutation with prominent paraspinal and proximal muscle involvement.* Neuromuscul Disord, 2013. **23**(7): p. 580-6.
205. Lefter, S., et al., *A novel MYH7 Leu1453pro mutation resulting in Laing distal myopathy in an Irish family.* Neuromuscul Disord, 2015. **25**(2): p. 155-60.
206. Sicko, R.J., et al., *Genetic Variants in Isolated Ebstein Anomaly Implicated in Myocardial Development Pathways.* PLoS One, 2016. **11**(10): p. e0165174.
207. Cullup, T., et al., *Mutations in MYH7 cause Multi-minicore Disease (MmD) with variable cardiac involvement.* Neuromuscul Disord, 2012. **22**(12): p. 1096-104.
208. Lamont, P.J., et al., *Novel mutations widen the phenotypic spectrum of slow skeletal/beta-cardiac myosin (MYH7) distal myopathy.* Hum Mutat, 2014. **35**(7): p. 868-79.
209. Fichna, J.P., et al., *Whole-exome sequencing identifies novel pathogenic mutations and putative phenotype-influencing variants in Polish limb-girdle muscular dystrophy patients.* Hum Genomics, 2018. **12**(1): p. 34.
210. Zeller, R., et al., *Large-scale mutation screening in patients with dilated or hypertrophic cardiomyopathy: a pilot study using DGGE.* J Mol Med (Berl), 2006. **84**(8): p. 682-91.
211. Meredith, C., et al., *Mutations in the slow skeletal muscle fiber myosin heavy chain gene (MYH7) cause laing early-onset distal myopathy (MPD1).* Am J Hum Genet, 2004. **75**(4): p. 703-8.
212. Kelly, M.A., et al., *Adaptation and validation of the ACMG/AMP variant classification framework for MYH7-associated inherited cardiomyopathies: recommendations by ClinGen's Inherited Cardiomyopathy Expert Panel.* Genet Med, 2018. **20**(3): p. 351-359.
213. Ferbert, A., et al., *Laing distal myopathy with a novel mutation in exon 34 of the MYH7 gene.* Neuromuscul Disord, 2016. **26**(9): p. 598-603.
214. Broendberg, A.K., et al., *Targeted next generation sequencing in a young population with suspected inherited malignant cardiac arrhythmias.* Eur J Hum Genet, 2018. **26**(3): p. 303-313.
215. Waldmuller, S., et al., *Low-density DNA microarrays are versatile tools to screen for known mutations in hypertrophic cardiomyopathy.* Hum Mutat, 2002. **19**(5): p. 560-9.
216. Carbonell-Corvillo, P., et al., *A novel MYH7 founder mutation causing Laing distal myopathy in Southern Spain.* Neuromuscul Disord, 2018. **28**(10): p. 828-836.
217. Beecroft, S.J., et al., *Recessive MYH7-related myopathy in two families.* Neuromuscul Disord, 2019. **29**(6): p. 456-467.
218. Chida, A., et al., *Prognostic predictive value of gene mutations in Japanese patients with hypertrophic cardiomyopathy.* Heart Vessels, 2017. **32**(6): p. 700-707.
219. Campuzano, O., et al., *Post-mortem genetic analysis in juvenile cases of sudden cardiac death.* Forensic Sci Int, 2014. **245**: p. 30-7.

220. Tajsharghi, H. and A. Oldfors, *Myosinopathies: pathology and mechanisms.* Acta Neuropathol, 2013. **125**(1): p. 3-18.
221. Clarke, N.F., et al., *A novel mutation expands the genetic and clinical spectrum of MYH7-related myopathies.* Neuromuscul Disord, 2013. **23**(5): p. 432-6.
222. Dabaj, I., et al., *Clinical and imaging hallmarks of the MYH7-related myopathy with severe axial involvement.* Muscle Nerve, 2018. **58**(2): p. 224-234.
223. Sarkozy, A., et al., *G.P.32 Progressive core myopathy with dilated cardiomyopathy, respiratory failure and severe scoliosis caused by a novel mutation in the MYH7 gene.* Neuromuscul Disord, 2012. **22**: p. 817-818.
224. Marston, S., et al., *OBSCN Mutations Associated with Dilated Cardiomyopathy and Haploinsufficiency.* PLoS One, 2015. **10**(9): p. e0138568.
225. Rupp, S., et al., *Genetic basis of hypertrophic cardiomyopathy in children.* Clin Res Cardiol, 2019. **108**(3): p. 282-289.
226. Liu, X.Y., et al., *A novel MYH7 mutation resulting in Laing distal myopathy in a Chinese family.* Chin Med J (Engl), 2019. **132**(7): p. 856-859.
227. Chen, K., et al., *Sarcomere variants in arrhythmogenic cardiomyopathy: Pathogenic factor or bystander?* Gene, 2019. **687**: p. 82-89.
228. Wang, L., et al., *Dual LQT1 and HCM phenotypes associated with tetrad heterozygous mutations in KCNQ1, MYH7, MYLK2, and TMEM70 genes in a three-generation Chinese family.* Europace, 2016. **18**(4): p. 602-9.
229. Romero, N.B., et al., *Autosomal dominant eccentric core disease caused by a heterozygous mutation in the MYH7 gene.* J Neurol Neurosurg Psychiatry, 2014. **85**(10): p. 1149-52.
230. Narula, N., et al., *Post-mortem Whole exome sequencing with gene-specific analysis for autopsy-negative sudden unexplained death in the young: a case series.* Pediatr Cardiol, 2015. **36**(4): p. 768-78.
231. Lee, D.D., et al., *Hypertrophic cardiomyopathy: a new mutation illustrates the need for family-centered care.* Pediatr Cardiol, 2014. **35**(8): p. 1474-7.
232. Dye, D.E., et al., *Novel slow-skeletal myosin (MYH7) mutation in the original myosin storage myopathy kindred.* Neuromuscul Disord, 2006. **16**(6): p. 357-60.
233. Xu, J., et al., *Investigation of Pathogenic Genes in Chinese sporadic Hypertrophic Cardiomyopathy Patients by Whole Exome Sequencing.* Sci Rep, 2015. **5**: p. 16609.
234. Forleo, C., et al., *Targeted next-generation sequencing detects novel gene-phenotype associations and expands the mutational spectrum in cardiomyopathies.* PLoS One, 2017. **12**(7): p. e0181842.
235. Brand, P., et al., *Distal myopathy with coexisting heterozygous TIA1 and MYH7 Variants.* Neuromuscul Disord, 2016. **26**(8): p. 511-5.
236. Yuceyar, N., et al., *Homozygous MYH7 R1820W mutation results in recessive myosin storage myopathy: scapuloperoneal and respiratory weakness with dilated cardiomyopathy.* Neuromuscul Disord, 2015. **25**(4): p. 340-4.
237. Tajsharghi, H., et al., *Myosin storage myopathy associated with a heterozygous missense mutation in MYH7.* Ann Neurol, 2003. **54**(4): p. 494-500.
238. Dal Ferro, M., et al., *Association between mutation status and left ventricular reverse remodelling in dilated cardiomyopathy.* Heart, 2017. **103**(21): p. 1704-1710.
239. Tajsharghi, H., et al., *Homozygous mutation in MYH7 in myosin storage myopathy and cardiomyopathy.* Neurology, 2007. **68**(12): p. 962.
240. Lin, Y., et al., *Applying High-Resolution Variant Classification to Cardiac Arrhythmogenic Gene Testing in a Demographically Diverse Cohort of Sudden Unexplained Deaths.* Circ Cardiovasc Genet, 2017. **10**(6).
241. Bohlega, S., et al., *Mutation of the slow myosin heavy chain rod domain underlies hyaline body myopathy.* Neurology, 2004. **62**(9): p. 1518-21.
242. Banfai, Z., et al., *Novel phenotypic variant in the MYH7 spectrum due to a stop-loss mutation in the C-terminal region: a case report.* BMC Med Genet, 2017. **18**(1): p. 105.
243. Ortolano, S., et al., *A novel MYH7 mutation links congenital fiber type disproportion and myosin storage myopathy.* Neuromuscul Disord, 2011. **21**(4): p. 254-62.

Supplemental Table 2: Missense mutations in MYH6 (α-cardiac myosin heavy chain) and their associated disease. HCM: hypertrophic cardiomyopathy. DCM: dilated cardiomyopathy. LVNC: Left Ventricular non-compaction. SUD: sudden unexplained death

Mutations	Myosin region	Disease	Reference
Ala12Val		HCM	[1]
Arg17Cys		DCM	[2]
Arg17His		Atrial septal defect	[3]
Arg17Leu		LVNC	[4]
Arg23His		HCM	[5]
Pro40Arg	SH3-like fold	Ventricular septal defect	[6]
Arg54Gln	SH3-like fold	long QY syndrome	[7]
Gly56Arg	SH3-like fold	HCM	[8]
Val71Ala	SH3-like fold	DCM	[9]
Glu98Lys		DCM	[10]
Val101Met		DCM	[9]
Arg108Cys		Congenital heart disease	[11]
Ala110Thr		DCM	[12]
Tyr115Asn		Hypoplastic left heart syndrome	[13]
Tyr162Cys		DCM	[12]
Ala199Ser	Loop-1	DCM	[9]
Arg204His	Loop-1	HCM	[14]
Asp208Asn	Loop-1	Atrioventricular septum defect	[15]
Ala230Pro		Congenital heart defects	[16]
Arg244His	Switch-1	Pulmonary hypertension	[17]
His252Gln		Congenital heart defects	[16]
Ile275Asn		DCM	[18]
Gln277His		Hypoplastic left heart syndrome	[13]
Pro308Leu		Pulmonary arterial hypertension	[17]
Ala336Gly		DCM	[10]
Met363Leu	Loop 4	HCM	[19]
Arg370Leu	Loop 4	LVNC	[20]
Gln373Pro	Loop 4	Congenital heart disease	[11]
Asp383Asn		Hypoplastic left heart syndrome	[13]
Ser385Leu		Hypoplastic left heart syndrome	[13]
Leu388Phe		Atrioventricular canal defects	[21]
Asp395GLu		SUD	[22]
Met436Val		Hypoplastic left heart syndrome	[13]
Arg443Cys		DCM	[10]
Arg443Pro		Hypoplastic left heart syndrome	[13]
His493Arg	Relay	SUD	[23]

Met494Ile	Relay	Left-ventricular outflow tract obstructions	[24]
Glu501Term		Congenital heart defects	[16]
Ile512Thr		Left ventricular obstruction with Shone complex	[21]
Glu526Lys	Activation loop	Pulmonary arterial hypertension	[17]
Cys539Arg	Helix-loop-helix	Atrial septal defect	[3]
Lys543Arg	Helix-loop-helix	Atrial septal defect	[3]
Arg568Cys	Loop-3	DCM	[18]
Gly585Cys		Pulmonary arterial hypertension	[17]
Gly585Ser		Left ventricular obstruction with Shone complex	[21]
Asp588Ala		Hypoplastic left heart syndrome	[25]
Asn678Ser		Congenital heart defects	[26]
Val700Met		Congenital heart defects	[16]
Ile704Asn		Hypoplastic left heart syndrome	[25]
Arg721Trp	Converter	Sick sinus syndrome	[27]
Ala769Val	Converter	Arrhythmogenic right ventricular cardiomyopathy	[28]
Arg795Gln	LCD	HCM	[29]
Arg795Trp	LCD	Hypoplastic left heart syndrome	[13]
Arg809Cys	LCD	HCM	[14]
Ile820Asn	LCD	Atrial septal defects	[30]
Pro830Leu	LCD	DCM	[31]
GLu846Asp	S2	DCM	[9]
Ala852Thr	S2	SUD	[22]
Arg860Cys	S2	Restrictive CM	[32]
Arg871His	S2	Cardiac arrest	[33]
Arg872Cys	S2	DCM	[10]
Ala936Val	S2	Atrioventricular septum defect	[15]
Lys942Term	S2	SUD young	[34]
Cys949Term	S2	Arrhythmogenic CM	[35]
Glu951Term	S2	Atrial septal defect	[23]
Asp955Val	S2	Bipolar	[36]
Ala964Ser	S2	Hypoplastic left heart syndrome	[13]
Glu1003Asp	S2	LVNC	[20]
Ala1004Ser	S2	DCM	[31]
Arg1047Cys	S2	DCM	[37]
Gln1065His	S2	HCM	[31]
Ile1068Thr	S2	Left ventricular obstruction with Shone complex	[21]
Lys1083Asn	S2	HCM	[32]
Ala1101Val	S2	Heart rate association with	[38]
Arg1116Cys	S2	DCM	[10]
Arg116Ser	S2	Congenital heart defects	[16]
Arg1143Gln	S2	DCM	[39]

Ser1149Arg	S2	DCM	[9]
Arg1151Gln	S2	Hypoplastic left heart syndrome	[13]
Arg1177Trp	S2	DCM	[18]
Glu1207Lys	S2	Hypoplastic left heart syndrome	[25]
Thr1253Met	LMM	DCM	[37]
Arg1279Term	LMM	Congenital heart defects	[40]
Glu1295Gln	LMM	Atrioventricular septum defect	[15]
Ala1298Val	LMM	Hypoplastic left heart syndrome	[13]
Ala1327Val	LMM	Atrioventricular canal defects	[21]
His1333Asn	LMM	Sudden unexplained death	[22]
Arg1339Trp	LMM	DCM	[41]
Ala1366Asp	LMM	Congenital heart defects	[16]
Thr1379Met	LMM	Hypoplastic left heart syndrome	[25]
Glu1389Lys	LMM	Sudden unexplained death	[22]
Arg1398Gln	LMM	Cardiac dysrhythmia	[42]
Val1406Met	LMM	DCM	[9]
Arg1422Leu	LMM	Pulmonary arterial hypertension	[17]
Asn1438Ile	LMM	Sudden unexplained death	[22]
Ala1440Pro	LMM	DCM	[18]
Ala1442Val	LMM	Abnormality of the mitochondrion	[43]
Ala1443Asp	LMM	Congenital heart defects	[16]
Glu1457Lys	LMM	DCM	[31]
Arg1502Gln	LMM	DCM	[18]
Glu1503Val	LMM	Hypoplastic left heart syndrome	[13]
Gln1543Lys	LMM	Sudden unexplained death	[22]
Phe1567Cys	LMM	DCM	[9]
Arg1576Gln	LMM	HCM	[44]
Glu1584Lys	LMM	Hypoplastic left heart syndrome	[13]
Arg1590Ser	LMM	Cardiac defects	[45]
Asn1591Ser	LMM	HCM	[46]
Arg1608Cys	LMM	Ventricular septal defect	[6]
Arg1610Cys	LMM	Atrial and ventricular septal defects	[21]
Lys1617Thr	LMM	LVNC	[20]
Glu1677GLn	LMM	Pulmonary arterial hypertension	[17]
Leu1690Arg	LMM	DCM	[41]
Arg1691Cys	LMM	HCM	[32]
Arg1699Trp	LMM	DCM	[39]
Ala1704Val	LMM	HCM	[47]
Ser1734Leu	LMM	Sudden unexplained death	[48]
Glu1754Term	LMM	Hypoplastic left heart syndrome	[13]
Met1766Val	LMM	DCM	[12]
Arg1820Gln	LMM	HCM	[49]

GLy1826Asp	LMM	Atrial septal defect	[6]
Lys1840Arg	LMM	Hypoplastic left heart syndrome	[13]
Glu1846Lys	LMM	HCM	[49]
Arg1865Gln	LMM	Congenital heart defects	[16]
Glu1885Lys	LMM	Wolff-parkinson-White syndrome	[50]
Ala1891Thr	LMM	Left ventricular obstruction with Shone complex	[21]
Arg1899Cys	LMM	DCM	[10]
Arg1899His	LMM	Left ventricular obstruction with Shone complex	[21]
Lys1932Term	LMM	Left ventricular obstruction with Shone complex	[21]

References

1. Guo, X., et al., *The clinical features, outcomes and genetic characteristics of hypertrophic cardiomyopathy patients with severe right ventricular hypertrophy.* PLoS One, 2017. **12**(3): p. e0174118.
2. Cuenca, S., et al., *Genetic basis of familial dilated cardiomyopathy patients undergoing heart transplantation.* J Heart Lung Transplant, 2016. **35**(5): p. 625-35.
3. Posch, M.G., et al., *Cardiac alpha-myosin (MYH6) is the predominant sarcomeric disease gene for familial atrial septal defects.* PLoS One, 2011. **6**(12): p. e28872.
4. Richard, P., et al., *Targeted panel sequencing in adult patients with left ventricular non-compaction reveals a large genetic heterogeneity.* Clin Genet, 2019. **95**(3): p. 356-367.
5. Bottillo, I., et al., *Molecular analysis of sarcomeric and non-sarcomeric genes in patients with hypertrophic cardiomyopathy.* Gene, 2016. **577**(2): p. 227-35.
6. Pulignani, S., et al., *Targeted Next-Generation Sequencing in Patients with Non-syndromic Congenital Heart Disease.* Pediatr Cardiol, 2018. **39**(4): p. 682-689.
7. Stepien-Wojno, M., et al., *Sudden cardiac arrest in patients without overt heart disease: a limited value of next generation sequencing.* Pol Arch Intern Med, 2018. **128**(12): p. 721-730.
8. Santos, S., et al., *High resolution melting: improvements in the genetic diagnosis of hypertrophic cardiomyopathy in a Portuguese cohort.* BMC Med Genet, 2012. **13**: p. 17.
9. Dal Ferro, M., et al., *Association between mutation status and left ventricular reverse remodelling in dilated cardiomyopathy.* Heart, 2017. **103**(21): p. 1704-1710.
10. Haas, J., et al., *Atlas of the clinical genetics of human dilated cardiomyopathy.* Eur Heart J, 2015. **36**(18): p. 1123-35a.
11. Homsy, J., et al., *De novo mutations in congenital heart disease with neurodevelopmental and other congenital anomalies.* Science, 2015. **350**(6265): p. 1262-6.
12. Walsh, R., et al., *Reassessment of Mendelian gene pathogenicity using 7,855 cardiomyopathy cases and 60,706 reference samples.* Genet Med, 2017. **19**(2): p. 192-203.
13. Tomita-Mitchell, A., et al., *Impact of MYH6 variants in hypoplastic left heart syndrome.* Physiol Genomics, 2016. **48**(12): p. 912-921.
14. Rubattu, S., et al., *A Next-Generation Sequencing Approach to Identify Gene Mutations in Early- and Late-Onset Hypertrophic Cardiomyopathy Patients of an Italian Cohort.* Int J Mol Sci, 2016. **17**(8).
15. Priest, J.R., et al., *De Novo and Rare Variants at Multiple Loci Support the Oligogenic Origins of Atrioventricular Septal Heart Defects.* PLoS Genet, 2016. **12**(4): p. e1005963.
16. Granados-Riveron, J.T., et al., *Alpha-cardiac myosin heavy chain (MYH6) mutations affecting myofibril formation are associated with congenital heart defects.* Hum Mol Genet, 2010. **19**(20): p. 4007-16.
17. Zhu, N., et al., *Rare variants in SOX17 are associated with pulmonary arterial hypertension with congenital heart disease.* Genome Med, 2018. **10**(1): p. 56.
18. Hershberger, R.E., et al., *Coding sequence rare variants identified in MYBPC3, MYH6, TPM1, TNNC1, and TNNI3 from 312 patients with familial or idiopathic dilated cardiomyopathy.* Circ Cardiovasc Genet, 2010. **3**(2): p. 155-61.
19. Zhao, Y., et al., *Identification of a novel hypertrophic cardiomyopathy-associated mutation using targeted next-generation sequencing.* Int J Mol Med, 2017. **40**(1): p. 121-129.
20. Miszalski-Jamka, K., et al., *Novel Genetic Triggers and Genotype-Phenotype Correlations in Patients With Left Ventricular Noncompaction.* Circ Cardiovasc Genet, 2017. **10**(4).

21. Jin, S.C., et al., *Contribution of rare inherited and de novo variants in 2,871 congenital heart disease probands.* Nat Genet, 2017. **49**(11): p. 1593-1601.
22. Lin, Y., et al., *Applying High-Resolution Variant Classification to Cardiac Arrhythmogenic Gene Testing in a Demographically Diverse Cohort of Sudden Unexplained Deaths.* Circ Cardiovasc Genet, 2017. **10**(6).
23. Liu, Y., et al., *Novel Genetic Variants of Sporadic Atrial Septal Defect (ASD) in a Chinese Population Identified by Whole-Exome Sequencing (WES).* Med Sci Monit, 2018. **24**: p. 1340-1358.
24. Preuss, C., et al., *Family Based Whole Exome Sequencing Reveals the Multifaceted Role of Notch Signaling in Congenital Heart Disease.* PLoS Genet, 2016. **12**(10): p. e1006335.
25. Theis, J.L., et al., *Recessive MYH6 Mutations in Hypoplastic Left Heart With Reduced Ejection Fraction.* Circ Cardiovasc Genet, 2015. **8**(4): p. 564-71.
26. Jia, Y., et al., *The diagnostic value of next generation sequencing in familial nonsyndromic congenital heart defects.* Am J Med Genet A, 2015. **167a**(8): p. 1822-9.
27. Holm, H., et al., *A rare variant in MYH6 is associated with high risk of sick sinus syndrome.* Nat Genet, 2011. **43**(4): p. 316-20.
28. Lu, C., et al., *Molecular analysis of inherited cardiomyopathy using next generation semiconductor sequencing technologies.* J Transl Med, 2018. **16**(1): p. 241.
29. Niimura, H., et al., *Sarcomere protein gene mutations in hypertrophic cardiomyopathy of the elderly.* Circulation, 2002. **105**(4): p. 446-51.
30. Ching, Y.H., et al., *Mutation in myosin heavy chain 6 causes atrial septal defect.* Nat Genet, 2005. **37**(4): p. 423-8.
31. Carniel, E., et al., *Alpha-myosin heavy chain: a sarcomeric gene associated with dilated and hypertrophic phenotypes of cardiomyopathy.* Circulation, 2005. **112**(1): p. 54-9.
32. Hayashi, T., et al., *Genetic background of Japanese patients with pediatric hypertrophic and restrictive cardiomyopathy.* J Hum Genet, 2018. **63**(9): p. 989-996.
33. Mellor, G., et al., *Genetic Testing in the Evaluation of Unexplained Cardiac Arrest: From the CASPER (Cardiac Arrest Survivors With Preserved Ejection Fraction Registry).* Circ Cardiovasc Genet, 2017. **10**(3).
34. Shanks, G.W., et al., *Importance of Variant Interpretation in Whole-Exome Molecular Autopsy: Population-Based Case Series.* Circulation, 2018. **137**(25): p. 2705-2715.
35. Chen, K., et al., *Sarcomere variants in arrhythmogenic cardiomyopathy: Pathogenic factor or bystander?* Gene, 2019. **687**: p. 82-89.
36. Kataoka, M., et al., *Exome sequencing for bipolar disorder points to roles of de novo loss-of-function and protein-altering mutations.* Mol Psychiatry, 2016. **21**(7): p. 885-93.
37. Zhao, Y., et al., *Targeted next-generation sequencing of candidate genes reveals novel mutations in patients with dilated cardiomyopathy.* Int J Mol Med, 2015. **36**(6): p. 1479-86.
38. Eijgelsheim, M., et al., *Genome-wide association analysis identifies multiple loci related to resting heart rate.* Hum Mol Genet, 2010. **19**(19): p. 3885-94.
39. Haskell, G.T., et al., *Whole Exome Sequencing Identifies Truncating Variants in Nuclear Envelope Genes in Patients With Cardiovascular Disease.* Circ Cardiovasc Genet, 2017. **10**(3).
40. Razmara, E. and M. Garshasbi, *Whole-exome sequencing identifies R1279X of MYH6 gene to be associated with congenital heart disease.* BMC Cardiovasc Disord, 2018. **18**(1): p. 137.
41. Gigli, M., et al., *Genetic Risk of Arrhythmic Phenotypes in Patients With Dilated Cardiomyopathy.* J Am Coll Cardiol, 2019. **74**(11): p. 1480-1490.
42. Gonzalez-Garay, M.L., et al., *Personalized genomic disease risk of volunteers.* Proc Natl Acad Sci U S A, 2013. **110**(42): p. 16957-62.
43. Retterer, K., et al., *Clinical application of whole-exome sequencing across clinical indications.* Genet Med, 2016. **18**(7): p. 696-704.
44. Xu, J., et al., *Investigation of Pathogenic Genes in Chinese sporadic Hypertrophic Cardiomyopathy Patients by Whole Exome Sequencing.* Sci Rep, 2015. **5**: p. 16609.
45. Hauser, N.S., et al., *Experience with genomic sequencing in pediatric patients with congenital cardiac defects in a large community hospital.* Mol Genet Genomic Med, 2018. **6**(2): p. 200-212.
46. Aljeaid, D., et al., *Prevalence of pathogenic and likely pathogenic variants in the RASopathy genes in patients who have had panel testing for cardiomyopathy.* Am J Med Genet A, 2019. **179**(4): p. 608-614.

47. Cecconi, M., et al., *Targeted next-generation sequencing helps to decipher the genetic and phenotypic heterogeneity of hypertrophic cardiomyopathy.* Int J Mol Med, 2016. **38**(4): p. 1111-24.
48. Campuzano, O., et al., *Post-mortem genetic analysis in juvenile cases of sudden cardiac death.* Forensic Sci Int, 2014. **245**: p. 30-7.
49. Lahrouchi, N., et al., *The yield of postmortem genetic testing in sudden death cases with structural findings at autopsy.* Eur J Hum Genet, 2020. **28**(1): p. 17-22.
50. Bowles, N.E., et al., *Exome analysis of a family with Wolff-Parkinson-White syndrome identifies a novel disease locus.* Am J Med Genet A, 2015. **167a**(12): p. 2975-84.

Supplemental Table 3: Missense mutations in MYH3 (embryonic myosin heavy chain) and their associated disease. SCT: spondylocarpotarsal synostosis syndrome

Val40Met	Arthrogryposis	[1]
Tyr47Term	SCT	[2]
Thr178Ile	Arthrogryposis, distal, type 2A	[3]
Gly184Ala	Arthrogryposis, distal, type 1	[4]
Ala234Thr	Arthrogryposis, distal 2B & myosin myopathy	[5]
Gly246Ala	Distal arthrogryposis syndrome 2b	[4]
Ser261Phe	Arthrogryposis, distal, type 2B	[3]
Phe287Val	Sheldon-Hall syndrome & vertebral fusions	[6]
Ser292Cys	Arthrogryposis, distal, type 2B	[3]
Thr333Arg	SCT	[7]
Leu340Gln	Distal arthrogryposis syndrome 2b	[4]
Glu375Lys	Arthrogryposis, distal, type 2B	[3]
Tyr387Cys	Arthrogryposis, distal, type 2A	[8]
Phe437Ile	Arthrogryposis, distal, type 1	[9]
Asp462Gly	Arthrogryposis, distal 2B & myosin myopathy	[5]
Phe466Cys	Distal arthrogryposis syndrome 2b	[4]
Glu498Gly	Arthrogryposis, distal, type 2A	[3]
Lys504Asn	Arthrogryposis, distal, type 1	[4]
Asp517Tyr	Arthrogryposis, distal, type 2B	[3]
Tyr583Ser	Arthrogryposis, distal, type 2A	[3]
Asp629Asn	Autism spectrum disorder	[10]
Phe645Cys	SCT	[11]
Arg672Cys	Arthrogryposis, distal, type 2A	[3]
Arg672His	Arthrogryposis, distal, type 2A	[3]
Gly769Val	Arthrogryposis, distal, type 2B	[3]
Val825Asp	Arthrogryposis, distal, type 2A	[3]
Lys838Glu	Arthrogryposis, distal, type 2B	[3]
Gln1075Pro	Pterygium syndrome	[12]
Arg1290His	Cleft lip and/or palate	[13]
Leu1320Pro	Arthrogryposis multiplex congenita with axoglial defects	[14]
Leu1344Pro	SCT	[7]
Asp1622Ala	Arthrogryposis, distal, type 2B	[3]
Ala1637Val	Arthrogryposis, distal, type 2B	[3]
Lys1652Arg	Developmental disorder	[15]

1. Xu, X., et al., *[Analysis of MYH3 gene variation and prenatal diagnosis for two pedigrees affected with congenital arthrogryposis].* Zhonghua Yi Xue Yi Chuan Xue Za Zhi, 2019. **36**(5): p. 447-450.
2. Cameron-Christie, S.R., et al., *Recessive SCT Due to Compound Heterozygosity for Variants in MYH3.* Am J Hum Genet, 2018. **102**(6): p. 1115-1125.

3. Toydemir, R.M., et al., *Mutations in embryonic myosin heavy chain (MYH3) cause Freeman-Sheldon syndrome and Sheldon-Hall syndrome.* Nat Genet, 2006. **38**(5): p. 561-5.
4. Beck, A.E., et al., *Spectrum of mutations that cause distal arthrogryposis types 1 and 2B.* Am J Med Genet A, 2013. **161a**(3): p. 550-5.
5. Tajsharghi, H., et al., *Embryonic myosin heavy-chain mutations cause distal arthrogryposis and developmental myosin myopathy that persists postnatally.* Arch Neurol, 2008. **65**(8): p. 1083-90.
6. Scala, M., et al., *A novel pathogenic MYH3 mutation in a child with Sheldon-Hall syndrome and vertebral fusions.* Am J Med Genet A, 2018. **176**(3): p. 663-667.
7. Carapito, R., et al., *Protein-altering MYH3 variants are associated with a spectrum of phenotypes extending to SCT.* Eur J Hum Genet, 2016. **24**(12): p. 1746-1751.
8. Beck, A.E., et al., *Genotype-phenotype relationships in Freeman-Sheldon syndrome.* Am J Med Genet A, 2014. **164a**(11): p. 2808-13.
9. Alvarado, D.M., et al., *Exome sequencing identifies an MYH3 mutation in a family with distal arthrogryposis type 1.* J Bone Joint Surg Am, 2011. **93**(11): p. 1045-50.
10. Iossifov, I., et al., *The contribution of de novo coding mutations to autism spectrum disorder.* Nature, 2014. **515**(7526): p. 216-21.
11. Zieba, J., et al., *A postnatal role for embryonic myosin revealed by MYH3 mutations that alter TGFbeta signaling and cause autosomal dominant spondylocarpotarsal synostosis.* Sci Rep, 2017. **7**: p. 41803.
12. Chong, J.X., et al., *Autosomal-Dominant Multiple Pterygium Syndrome Is Caused by Mutations in MYH3.* Am J Hum Genet, 2015. **96**(5): p. 841-9.
13. Pengelly, R.J., et al., *Deleterious coding variants in multi-case families with non-syndromic cleft lip and/or palate phenotypes.* Sci Rep, 2016. **6**: p. 30457.
14. Laquerriere, A., et al., *Mutations in CNTNAP1 and ADCY6 are responsible for severe arthrogryposis multiplex congenita with axoglial defects.* Hum Mol Genet, 2014. **23**(9): p. 2279-89.
15. *Prevalence and architecture of de novo mutations in developmental disorders.* Nature, 2017. **542**(7642): p. 433-438.

Supplemental Table 4: Missense mutations in MYH2 (MyHC-2a) and their associated disease.

Thr178Ile	Myopathy with external ophthalmoplegia	[1]
Ala236Thr	Myopathy with external ophthalmoplegia	[1]
Arg246His	Myopathy, MYH2-related	[2]
Ala387Val	Myopathy, proximal with ophthalmoplegia	[3]
Val423Met	Proximal myopathy	[4]
Arg445Cys	Myopathy with external ophthalmoplegia	[1]
Glu500Term	Myopathy with external ophthalmoplegia	[5]
Phe516Val	Congenital myopathy	[6]
Met531Thr	Myopathy with external ophthalmoplegia	[1]
His672Tyr	Pulmonary arterial hypertension	[7]
Glu706Lys	Inclusion body myopathy	[8]
Asp756Asn	Myopathy	[9]
Arg783Term	Myopathy, early onset	[10]
Leu802Term	Myopathy, early onset	[10]
Val805Ala	Inclusion body myositis	[11]
Lys890Ile	Pulmonary arterial hypertension	[7]
Val970Ile	Inclusion body myopathy	[12]
Ser1043Ala	Inclusion body myositis	[13]
Leu1061Val	Inclusion body myopathy	[12]
Gln1111Term	Congenital myopathy	[6]
Arg1218Term	Autism spectrum disorder	[14]
Lys1311Gln	Pulmonary arterial hypertension	[7]
Leu1420Phe	Neuromuscular disorder	[15]
Glu1681Lys	Inclusion body myositis	[13]
Leu1870Pro	Myopathy, MYH2-related	[16]
Leu1877Pro	Myopathy, distal and proximal with complete ophthalmoplegia	[17]

References

1. Tajsharghi, H., et al., *Recessive myosin myopathy with external ophthalmoplegia associated with MYH2 mutations.* Eur J Hum Genet, 2014. **22**(6): p. 801-8.
2. Findlay, A.R., et al., *Homozygous recessive MYH2 mutation mimicking dominant MYH2 associated myopathy.* Neuromuscul Disord, 2018. **28**(8): p. 675-679.
3. Posey, J.E., et al., *Resolution of Disease Phenotypes Resulting from Multilocus Genomic Variation.* N Engl J Med, 2017. **376**(1): p. 21-31.
4. Krenn, M., et al., *Genotype-guided diagnostic reassessment after exome sequencing in neuromuscular disorders: experiences with a two-step approach.* Eur J Neurol, 2020. **27**(1): p. 51-61.
5. Hernandez-Lain, A., et al., *Myosin myopathy with external ophthalmoplegia associated with a novel homozygous mutation in MYH2.* Muscle Nerve, 2017. **55**(2): p. E8-e10.
6. Nicolau, S., et al., *Congenital myopathies in the adult neuromuscular clinic: Diagnostic challenges and pitfalls.* Neurol Genet, 2019. **5**(4): p. e341.

7. Zhu, N., et al., *Rare variants in SOX17 are associated with pulmonary arterial hypertension with congenital heart disease.* Genome Med, 2018. **10**(1): p. 56.
8. Martinsson, T., et al., *Autosomal dominant myopathy: missense mutation (Glu-706 --> Lys) in the myosin heavy chain IIa gene.* Proc Natl Acad Sci U S A, 2000. **97**(26): p. 14614-9.
9. Chen, N., et al., *Clinical remission of myopathy with MYH2 deficiency after precision medicine-developed rehabilitation: a case report.* Am J Transl Res, 2018. **10**(11): p. 3827-3832.
10. Tajsharghi, H., et al., *Human disease caused by loss of fast IIa myosin heavy chain due to recessive MYH2 mutations.* Brain, 2010. **133**(Pt 5): p. 1451-9.
11. Cai, H., et al., *Clinical, pathological, and genetic mutation analysis of sporadic inclusion body myositis in Japanese people.* J Neurol, 2012. **259**(9): p. 1913-22.
12. Tajsharghi, H., et al., *Mutations and sequence variation in the human myosin heavy chain IIa gene (MYH2).* Eur J Hum Genet, 2005. **13**(5): p. 617-22.
13. Weihl, C.C., et al., *Targeted sequencing and identification of genetic variants in sporadic inclusion body myositis.* Neuromuscul Disord, 2015. **25**(4): p. 289-96.
14. Iossifov, I., et al., *The contribution of de novo coding mutations to autism spectrum disorder.* Nature, 2014. **515**(7526): p. 216-21.
15. Tian, X., et al., *Expanding genotype/phenotype of neuromuscular diseases by comprehensive target capture/NGS.* Neurol Genet, 2015. **1**(2): p. e14.
16. D'Amico, A., et al., *A new de novo missense mutation in MYH2 expands clinical and genetic findings in hereditary myosin myopathies.* Neuromuscul Disord, 2013. **23**(5): p. 437-40.
17. Cabrera-Serrano, M., et al., *Adult onset distal and proximal myopathy with complete ophthalmoplegia associated with a novel de novo p.(Leu1877Pro) mutation in MYH2.* Clin Genet, 2015. **88**(6): p. 573-8.
